# Time- and site-resolved kinetic NMR for real-time monitoring of off-equilibrium reactions by 2D spectrotemporal correlations

**DOI:** 10.1038/s41467-022-28304-w

**Published:** 2022-02-11

**Authors:** Michael J. Jaroszewicz, Mengxiao Liu, Jihyun Kim, Guannan Zhang, Yaewon Kim, Christian Hilty, Lucio Frydman

**Affiliations:** 1 Department of Chemical and Biological Physics, Weizmann Institute of Science, Rehovot, Israel; 2 Chemistry Department, Texas A&M University, College Station, TX USA

**Keywords:** Reaction kinetics and dynamics, Solution-state NMR

## Abstract

Nuclear magnetic resonance (NMR) spectroscopy provides detailed information about dynamic processes through line-shape changes, which are traditionally limited to equilibrium conditions. However, a wealth of information is available by studying chemical reactions under off-equilibrium conditions—*e.g*., in states that arise upon mixing reactants that subsequently undergo chemical changes—and in monitoring the reactants and products in real time. Herein, we propose and demonstrate a time-resolved kinetic NMR experiment that combines rapid mixing techniques, continuous flow, and single-scan spectroscopic imaging methods, leading in unison to a 2D spectrotemporal NMR correlation that provides high-quality kinetic information of off-equilibrium chemical reactions. These kinetic 2D NMR spectra possess a high-resolution spectral dimension revealing the individual chemical sites, correlated with a time-independent, steady-state spatial axis that delivers information concerning temporal changes along the reaction coordinate. A comprehensive description of the kinetic, spectroscopic, and experimental features associated with these spectrotemporal NMR analyses is presented. Experimental demonstrations are carried out using an enzymatically catalyzed reaction leading to site- and time-resolved kinetic NMR data, that are in excellent agreement with control experiments and literature values.

## Abstract (web summary)

Time-resolved NMR spectra provide unique structural and dynamical information, but their measurement in systems undergoing chemical reactions is challenging. Here the authors, combining single-scan spectroscopic imaging, rapid mixing and continuous flow techniques, obtain chemically resolved snapshots of a reacting system throughout the reaction coordinate.

## Introduction

Nuclear Magnetic Resonance (NMR) spectroscopy is capable of studying dynamic processes with atomic-level resolution over a large range of time scales, owing to the extreme sensitivity of the nuclear spin interactions to changes in the surrounding atomic environment. For example fast dynamic processes occurring on 10^−7^–10^−3^ s time scales can be studied with suitable relaxation-time measurements^1^, while slower chemical exchanges in the 10^−3^–10^0^ s scales can be monitored by analyzing variations in the resulting NMR spectral line shapes^2,3^. Both of these cases are usually studied under equilibrium conditions, whereby dynamic fluctuations happen but chemical species remain under steady-state concentrations. A wealth of information, however, can also be garnered by studying dynamic processes that begin from an off-equilibrium state, and monitoring the progression of chemical kinetics as it proceeds towards equilibrium in real time. So-called stopped-flow NMR techniques have proven particularly effective at studying off-equilibrium chemical reactions, without suffering from deleterious spectral artifacts that may otherwise arise from the transient formation and decay of species^4–7^. Stopped-flow NMR has thus been used for studying chemical reactions in polymer and in biomolecular chemistry (*e.g*., protein and nucleic acid folding)^8^; stopped-flow NMR has also been combined with nuclear hyperpolarization methods, which among other benefits allows for the facile detection of unreceptive nuclei^9^. Despite their advantages, stopped-flow NMR methods are limited by the mandatory relaxation-delay and dead-time periods needed for spin polarization, mixing, and sample stabilization, as well as the necessity to acquire data from multiple sample batches: all these demands complicate the method, and decrease its capability to analyze the reaction coordinate. There exists, however, another method for monitoring chemical reactions with NMR that can also provide information with respect to the temporal characteristics of reactions processes, which involves the continuous flow of reagents through the NMR probehead^10–14^. One such possibility relies on a continuous flow reactor, in which the flow rate of reactants is systematically changed; different stages in the reaction can then come into view of the detector, even if the method is challenged by short residence times and requires an ample supply of reactants. Alternatively, experiments can monitor spectra at constant flow rates but at one or more instants following reagent mixing. Even further information with respect to the spatial—and hence the temporal—distribution of reaction processes could arise if they were monitored as a function of position within the NMR coil. Based on this premise, this work introduces and develops a time-resolved kinetic NMR experiment that combines rapid-mixing and continuous-flow methodologies, with single-scan spectroscopic imaging principles. The result is a novel type of 2D spectrotemporal NMR correlation experiment that is capable of providing high-quality, site-resolved kinetic insight about chemical reactions. The principles underlying these time- and site-resolved kinetic NMR experiments are discussed in the subsequent sections, including experimental demonstrations that monitored the depletion and formation of ^1^H methyl resonances evolving from an enzymatically-catalyzed hydrolysis reaction with millisecond time resolution.

## Results

### Chemical kinetics from 2D spectrotemporal NMR correlations: overall principles

**Fig. 1:**
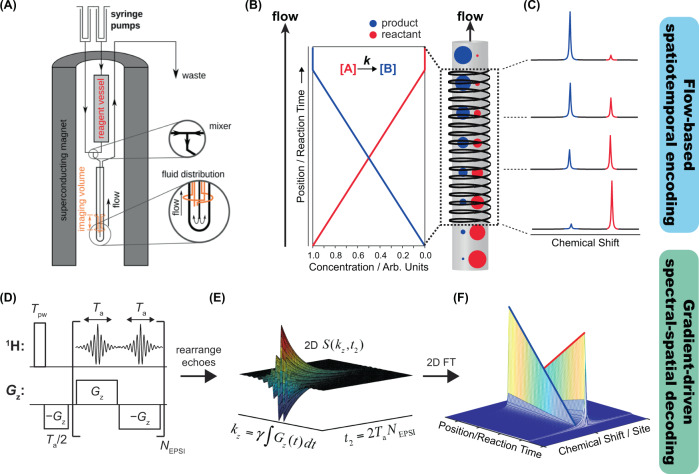
Time-resolved kinetic 2D NMR spectrotemporal correlations arising by combining flow-based spatiotemporal encoding and gradient-driven spectroscopic imaging decoding. **A** Experimental setup for encoding off-equilibrium kinetics using rapid-mixing and continuous plug-like flow, incorporating a syringe-driven apparatus integrated with a gradient-equipped NMR flow probehead and high-field NMR magnet. **B** Idealized plot of the time-dependent concentrations evolving from a reaction mixture characterized by a zero-order rate law, in which the depletion and formation of the reactant and product are linear functions in time. As this reaction mixture flows through the NMR coil, the plug-like flow encodes each position along the +*z* direction with a unique, steady-state proportion of reactant and product, as schematically indicated by the size of the red and blue circles, respectively. **C** Each position in space is therefore encoded with a unique NMR spectrum that corresponds to a distinct and definite moment along the reaction process. **D** The echo-planar spectroscopic imaging (EPSI) pulse sequence is used to retrieve and decode the spatially-encoded kinetic information, by resolving NMR spectra as a function of position using an oscillating bipolar gradient-echo train. **E**, **F** Post-processing of the 1D EPSI time-domain dataset followed by Fourier transformation with respect to the imaging- and time-domain variables *k*_z_ and *t*_2_, respectively, reveal the spatiotemporally-encoded kinetic NMR spectra.

At the heart of the spectrally-resolved kinetic NMR experiment hereby discussed is the co-application of rapid-mixing flow methodologies with ultrafast spatial-spectral imaging schemes. To understand how the combined use of these two modalities allows for the real-time observation of off-equilibrium reactions, consider a situation in which a chemical reaction is initiated by the rapid mixing of reagents. These reagents then flow with uniform plug-like velocity through a vertical NMR tube with both ends open. The tube is wrapped by an observation (*e.g*. Helmholtz) coil, where flow occurs in the *z* direction parallel to the external field, while the coil is located downstream from the point of mixing (Fig. 1A). Suppose as well that the spin magnetization of at least one of the reactants is pre-polarized, and that the flow is calibrated in such a manner that regions located near the bottom and top of the NMR coil contain predominantly reactants and products, respectively; *i.e*., concentration changes and intermediate transient reaction species occur at locations within the NMR coil’s field of view. Then, under conditions of stable, continuous, and plug-like flow, each point located at a defined distance away from the point of mixing will contain a unique set of chemical species that correspond to a particular time point along the reaction coordinate (Fig. 1B). This in turn will give rise to a unique, *z*-dependent NMR spectrum, whereby the unidirectional flow maps in a one-to-one fashion the reaction coordinate of the kinetic process onto the *z* spatial dimension of the NMR coil. Thus, even though the NMR-emitting spins are changing with respect to time due to the kinetics and varying their positions due to the flow, each *z* position will yield a distinct, steady NMR spectrum that corresponds to a definite moment in the reaction process (Fig. 1C). Given such a scenario, it is possible to retrieve temporally-resolved kinetic information about how the reaction affects individual chemical sites, using a 2D chemical shift imaging approach^15–17^ in which gradient-based imaging manipulations resolve, while maintaining chemical site resolution, NMR spectra arising along distinct positions of the *z* spatial axis. While numerous spectroscopic imaging approaches can do this, speed is also of the essence in this particular case, as the syringe-driven kinetic steady state cannot be maintained indefinitely. Consequently, we decided to employ the echo-planar spectroscopic imaging (EPSI) readout block (Fig. 1D)^18–20^, which delivers 2D spatial/spectral NMR datasets in a single scan, correlating the spins’ position using a single-axis gradient (in this case, the direction of the flow along the *z* axis) in one dimension, with chemical shifts along the orthogonal dimension. The EPSI signal readout occurs while oscillating a bipolar gradient that repeatedly de-phases and re-phases the transverse spin magnetization, resulting in a series of gradient echoes modulated in time by the chemical shift evolution (and by *J*-couplings, here disregarded). Achieving the desired spectrally-resolved 1D images is accomplished by post-processing, which first involves splicing the 1D EPSI signal (FID) into a 2D matrix, whereby each gradient echo is placed in a single row and indexed as a function of a time variable *t*_2_ (Fig. 1E). In this way, the *z* spatial information is encoded along rows defined by a wavenumber *k*_z_, while *t*_2_ encodes down each column the chemical shifts – whose evolution is unaffected by the oscillating gradients. After such rearrangement, which may include appropriate alignment of the even and odd gradient echoes, 2D Fourier transformation with respect to *k*_z_ and *t*_2_ reveals the spectrally-resolved 1D spatial profiles for each chemical site. If the time *T*_a_ required to collect each gradient echo (≈100–500 µs) is small with respect to the timescale of the kinetic process, this 2D dataset then reflects the underlying kinetics affecting each chemical site over the course of the reaction (Fig. 1F).

With these principles as background, the sections that follow delve further into the details of this spectrotemporal 2D correlation approach to monitor chemical kinetics in real time. Proof of the experimental feasibility and validations of this new kinetic NMR methodology are presented in the following Paragraph, which describes the real-time monitoring of the time-dependent depletion and formation of two methyl proton resonances, corresponding respectively to single reactant and product species evolving from an enzymatically-catalyzed, zero-order, off-equilibrium reaction. Then, subsequent Paragraphs within the main and supplementary texts, present a theoretical analysis of the underlying spin dynamics involved in these kinetic spectral imaging experiments. To facilitate a better understanding of the ensuing features of the method, the Supplementary Information treats three models that, in increasing order of complexity, show how different facets of the rapid mixing, plug-like flow, and oscillating imaging gradients, end up providing a tool for chemical kinetics. Analytical and numerical derivations are thus compared among themselves and with experiments, and the application of these kinetic NMR methods towards investigating a wider range of rapid off-equilibrium chemical reactions is discussed. A survey of the current limitations and the challenges affecting these techniques, and potential strategies for how they might be overcome and remedied, are also provided.

### 2D spectrotemporal NMR Correlations: experimental tests using an enzymatically-catalyzed reaction

**Fig. 2:**
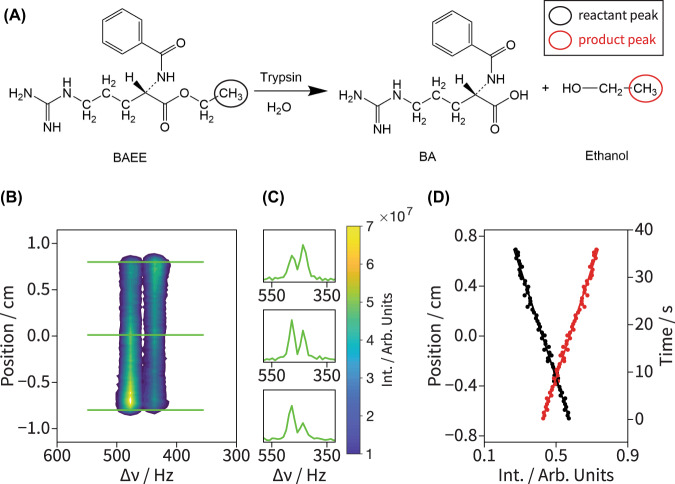
Kinetic analysis of an enzymatically-catalyzed reaction recorded under continuous flow, off-equilibrium conditions *via* 2D spectrotemporal correlations. **A** Chemical structures of the substrate *N*-α-benzoyl arginine ethyl ester (BAEE), and reaction products benzoyl arginine (BA) and ethanol. The CH_3_ groups monitored in the experiments are circled in black and red for the reactant and product, respectively. **B** ^1^H EPSI NMR spectrum of 10 mM BAEE reacting with 7.5 µM trypsin using the pulse sequence in Fig. 1D (*T*_a_ = 0.5 ms, *N*_EPSI_ = 100, Δ*z* = 230 μm, *v* = 0.33 mL/min, Δ*t*_R_ = 580 ms); **C** green plots show three spectral cross sections taken at the indicated line positions (also green). **D** Integrated spectral intensities (circles) of the reactant (black circles) and product (red circles) resonances plotted against position within the coil. The known rate of flow permits translating positions into reaction times; kinetic fitting of the integrated intensities as discussed in the main text leads to the red and black solid lines, which yielded *k*_cat_ = 11.8 ± 0.5 s^−1^ and *t*_0_ = 48 ± 3 s for three consecutive acquisitions. A relaxation delay of 5 s and 64 scans were used in these experiments.

The enzymatically-catalyzed hydrolysis of *N*-α-benzoyl arginine ethyl ester (BAEE) was chosen as a test reaction^9,21^ for the method here described (Fig. 2A). Supporting Information S1 and the Methods section provide details on how the continuous-flow apparatus was built and operated. In it, a continuous flow of the substrate (BAEE) was provided through a pre-polarization chamber feeding into the first channel of the mixer, while a solution with the catalyst (the enzyme trypsin) flowed through a second channel without pre-polarization. This resulted in the selective polarization of BAEE’s ^1^Hs prior to the mixing. These flows were driven by a syringe pump at a controllable flow rate, and included a return channel for clearing out the products of the reaction outside the NMR probe’s field of view. The mixed, reacting solution rapidly flowed through a narrow central inlet tubing to the bottom of an NMR tube. Subsequent to this sudden inlet flow, the fluid proceeded in a slower, upwards flow, with a velocity that was adjusted for the reaction so as to reach near completion within the sensitive region along the *z*-axis of the NMR coil. This construction allows the device to be top-loaded into a conventional 5 mm NMR probe without requiring special flow-through capabilities. Because the central inlet tubing (0.5 mm inner diameter) was much narrower than the larger return path (4.2 mm inner diameter), the resulting 70-fold volume difference between the down- and up-flowing solutions allowed us to neglect the former’s signal contributions. ^1^H NMR spectra of the flowing reaction mixture were acquired using 2D EPSI and slice-selective imaging pulse sequences, in which the proton methyl resonance belonging to BAEE and the reaction product ethanol (circled in Fig. 2A) were targeted using selective excitations. See Methods below for additional details on the experimental setup.

Figure 2B–D shows experimental 2D ^1^H EPSI data acquired for the flowing reaction, executed with 10 mM BAEE and 7.5 µM trypsin. The depletion of the BAEE reactant peak (*ν*_iso_ = 476 Hz; 1.19 ppm) and build-up of the ethanol product peak (*ν*_iso_ = 437 Hz; 1.09 ppm) are clearly visible in both the 2D spatial-spectral contours (Fig. 2B), as well as in 1D cross sections taken at the indicated *z* positions from the contour plot (horizontal green lines). Also evident is a certain loss in spectral resolution leading to a blurring of the *J*-coupling structures, which we ascribe to the onset of turbulences leading to *T*_*2*_*-*like effects under the action of the oscillating gradients. Under these experimental conditions (*N*_EPSI_ = 100, *T*_a_ = 0.5 ms, *G*_a_ = 20.5 G/cm), the spatial resolution of the EPSI readout is Δ*z* = *ca*. 230 μm, which when factored with the linear flow rate *ν* = 390 μm/s, results in an overall temporal resolution along the reaction coordinate of Δ*t*_R_ = *ca*. 580 ms. When coupled to the plug-flow assumption and to a knowledge of the flow rate, integration of the spectrally-resolved ^1^H peaks that EPSI provides in a position-dependent fashion, yields the state of the reaction at different moments in time (Fig. 2D). To extract the catalysis rate *k*_cat_ that controls the reaction we realize that, under the conditions assayed, the product formation rate is ultimately proportional to the total enzyme concentration: $\frac{d[P]}{{dt}}={k}_{{{{{{\rm{cat}}}}}}}{[E]}_{0}$. Determining *k*_cat_ is possible by integrating this equation, while realizing that the total amount of reactant and product at any given time, [R] and [P], will equal the total initial concentration [R]_0_ of BAEE. Assuming that the position-dependent normalized integration values for the signals of the reactant peak *S*_R_ (black dots) and product peak *S*_P_ (red dots) will be proportional to these concentrations, the position-dependence of the observed resonances can be expressed as:

1 $${S}_{{{{{{\rm{R}}}}}}}(z)=\frac{1}{{[R]}_{0}}\left({[R]}_{0}-{k}_{{{{{{\rm{cat}}}}}}}\cdot {\left[E\right]}_{0}\cdot (z+z_{0})/{v}\right)$$

2 $${S}_{{{{{{\rm{P}}}}}}}\left(z\right)=\frac{1}{{\left[R\right]}_{0}}\left({k}_{{{{{{\rm{cat}}}}}}}\cdot {\left[E\right]}_{0}\cdot (z+z_{0})/{v}\right),$$

where ${\left[E\right]}_{0}$, is the initial trypsin concentration, and *z*/*v* provides the reaction time *t*. *t*_0_ = *z*_0_/*v* thus defines the residence time in the “dead volume” occupying the latent space between the point of mixing and the NMR coil, predominantly at the bottom of the NMR tube. After three consecutive EPSI NMR measurements, the average *k*_cat_ and *t*_0_ values were determined to be *k*_cat_ = 11.8 ± 0.5 s^−1^ and *t*_0_ = 48 ± 3 s; given the known flow velocity *v,* the latter corresponds to a dead volume of *ca*. 240 μL. This experimentally measured *k*_cat_ value is in excellent agreement with previously reported values for this chemical reaction^9,21^.

**Fig. 3:**
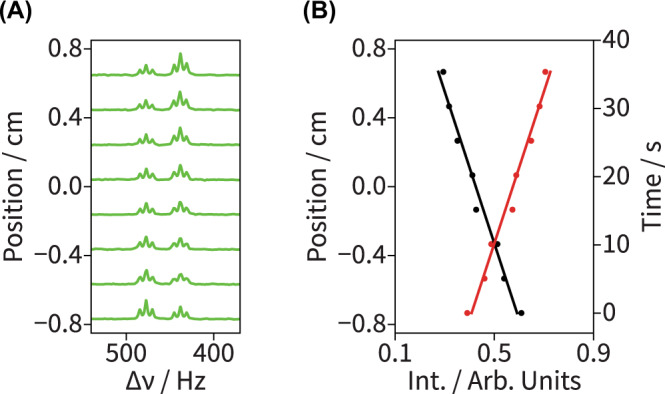
1D Slice-selective NMR spectra of the enzymatically-catalyzed hydrolysis reaction described in Fig. 2. **A** Slice-selective (2 mm) ^1^H NMR spectra of the methyl region acquired at 8 distinct positions along the NMR coil, collected under similar experimental conditions as those in Fig. 2, but utilizing a 1 second acquisition time. The improved resolution reveals the *J*-coupling of the sites. **B** Integrated signal intensities for the reactant (black circles) and product (red circles) plotted as a function of both position and reaction time against the lines of best fit from the kinetic analysis (*k*_cat_ = 12.0 s^−1^ and *t*_0_ = 45 s).

In addition to these EPSI-based kinetic NMR experiments, a series of slice-selective control experiments were performed (Fig. 3). In these, several 1D position-dependent (*i.e*., time-resolved) NMR spectra were collected under similar experimental conditions as described in Fig. 2, except that the NMR signals (FIDs) were acquired without gradients for longer times (200 ms *vs*. 1000 ms, with the former time given by limitations in the duration over which the gradients could be applied); this led to a better spectral resolution (see Supplementary Table 2 for a summary on the sensivitiy and resolution afforded by each method). A total of 8 consecutive slice-selective 1D ^1^H NMR spectra were recorded at different *z* positions throughout the sensitive region of the NMR coil (Fig. 3A), and the corresponding integrated intensities for both the reactant and product peaks (black and red curves, respectively) were calculated and fit in exactly the same manner as in the EPSI measurements (Fig. 3B). In this case, both *k*_cat_ and *t*_0_ were measured to be 12 s^−1^ and 45 s, respectively; again, in very good agreement with the kinetic parameters obtained with EPSI.

**Fig. 4:**
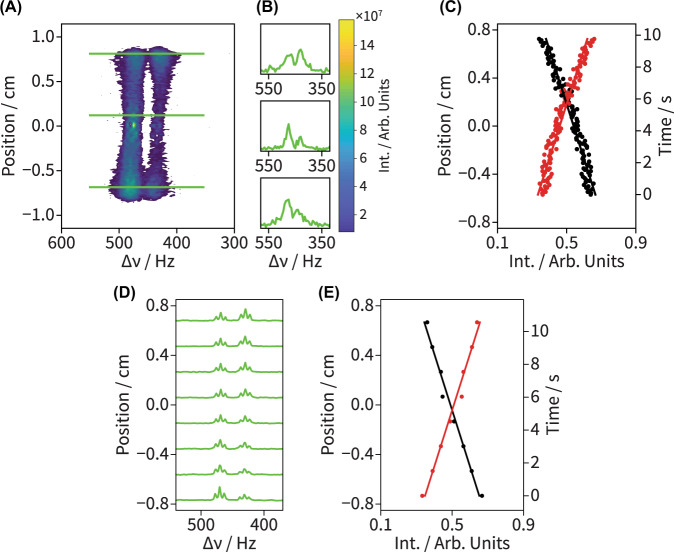
High-resolution (Δ*t*_R_ = 86 ms) time-resolved kinetic NMR spectra of an enzymatically-catalyzed hydrolysis reaction in off-equilibrium. **A** ^1^H EPSI NMR spectrum and **B** associated spectral cross sections acquired with a reaction-time resolution of Δ*t*_R_ = 86 ms under conditions of continuous flow for a reaction mixture of 25 µM trypsin and 10 mM BAEE. The following experimental parameters were used in the collection of **A**: *N*_EPSI_ = 100, *T*_a_ = 1 ms, *G*_z_ = 20.5 G/cm, time-domain data size *TD* = 80,000 (200 complex pts/echo), and *v* = 1200 μm/s (1 mL/min). 240 scans were collected with recycle delay *D1* = 5 s. **C** Integrated signal intensities for the reactant (black circles) and product (red circles) plotted as functions of both position and reaction time against the lines of best fit from the kinetic analysis (solid lines): *k*_cat_ = 11.7 s^−1^ and *t*_0_ = 11 s. **D** ^1^H slice-selective pulse-acquire NMR spectra acquired at 8 distinct positions across the NMR coil and under similar experimental conditions as those in **A**–**C**: 25 µM trypsin, 10 mM BAEE, and the flow rate *v* = 1 mL/min. The kinetic fitting results obtained from these data are: *k*_cat_ = 11.7 s^−1^ and *t*_0_ = 12 s.

A feature of the kinetic NMR method introduced in Fig. 1 is its capability to control the time resolution used to probe the reaction coordinate in a simple, programmable manner. For many time-resolved kinetic NMR techniques this time resolution is often dictated by factors requiring major setup changes—like modifications in the fluid path length or in the rate at which reactants flow. For the spatially-encoded NMR spectra revealed by 2D spectrotemporal correlations, kinetic time resolution is ultimately governed, for a given flow rate, by the spatial resolution of the EPSI imaging readout—an easily tunable and controllable parameter. To demonstrate this added flexibility, Fig. 4 shows how the time resolution can be increased by a factor of *ca*. 6 *vs*. that in Fig. 2, by increasing both the flow rate by a factor of 3.3× (*v* ≈ 1 mL/min), and the EPSI gradient readout spatial resolution by a factor of 2× (*T*_a_ = 1 ms and Δ*z* = 115 μm). Under these experimental conditions, a reaction-time resolution of *ca*. 86 ms was achieved. Note that this time resolution is shorter than the total time required to collect the entire EPSI gradient echo train, which lasted 200 ms in total (*i.e*., *N*_EPSI_ = 100 for *T*_a_ = 1 ms). This added flexibility for monitoring kinetic processes with high temporal resolution comes at a price, however, which is evidenced by the *ca*. 3× reduction in signal-to-noise (SNR) for Fig. 4*vs*. Fig. 2. This results from the combined effects of the increased flow rate and of the higher spatial resolution of the gradient readout. Also noticeable is a further deterioration of the spectral resolution, as a result of the signal decay brought about by turbulences acting in the presence of stronger field gradients. Despite these SNR and resolution deteriorations, it is still possible to achieve similar kinetic fits as those ascertained in Fig. 2: three independent measurements here yielded *k*_cat_ = 11.7 ± 0.3 s^−1^ and *t*_0_ = 12 ± 1 s. As before, 1D slice-selective pulse-acquire measurements carried out under the same experimental conditions yield virtually identical kinetic parameters (Fig. 4D, E). Supplementary Note 2 and Supplementary Figs. 4–6 contained therein provide a more in-depth analysis of the combined effects of the imaging spatial resolution and flow rate on the overall SNR.

### The spatio-temporal encoding and spectral-spatial decoding processes

The information of 2D spectrotemporal kinetic correlations arises from the simultaneous action of flow-based and gradient-based encoding and decoding schemes. As such they exhibit, in addition to chemical kinetic information, dependencies on both the flow and imaging characteristics of the experiment. An analysis of these characteristics is thus justified; the present paragraph examines how the chemical kinetics interacts with the flow and with the specific EPSI experiment, to govern the spin evolution eventually leading to 2D spectrotemporal NMR correlation spectra. We consider a continuous plug-like flow of an off-equilibrium reaction mixture that occurs before and during the acquisition of an FID, which can be excited by a single resonant radio-frequency (RF) pulse or by an EPSI readout. It is also assumed that the chemical reaction is described by an irreversible zero-order rate law ($A\mathop{\to }\limits^{k}B$), that is instantaneously initiated in a reaction vessel located immediately upstream from the NMR coil; *i.e*., for simplicity we assume there is no latent volume between the point at which the reaction is initiated and the entrance into the NMR coil. At the coil thus enters only the reactant **A**, with the product **B** forming progressively down the length of the coil. A linear flow at rate *v* gives a distribution of **A** and **B** longitudinal magnetization that is a function of both position and time, which exists in a dynamic equilibrium for all fixed positions *z* within the NMR coil when *vt* > *L*_z_, where *t* is the elapsed time post-mixing, and *L*_z_ is the length of the NMR coil. Describing the spin dynamics of **A** and **B** under these combined conditions of plug-like flow and zero-order chemical kinetics is easier if first the dynamics of **A** and **B** are determined in the absence of flow. A description for such a case is presented in Supplementary Note 3. Denoting *S*_A_(*t*) and *S*_B_(*t*) the signals then emitted by **A** and **B** (still in the absence of flow or field gradients) after a hard-pulse excitation of the entire sample at a time *t* = 0 coinciding with the time at which the reaction is initiated, this Supplement shows that

3 $${S}_{{{{{{\rm{A}}}}}}}(t)\propto \left\{\begin{array}{cc}({A}_{0}-{kt})\times \exp(\lambda_{{{{{{\rm{A}}}}}}}t), & {t} \, < \, {t}_{\max} \\ \hfill 0, & t \ge {t}_{\max}\end{array}\right.$$

and

4 $${S}_{{{{{{\rm{B}}}}}}}\left(t\right)\propto \left\{\begin{array}{cc}k\frac{{{{{{\rm{exp }}}}}}\left({\lambda }_{{{{{{\rm{A}}}}}}}t\right)-{{{{{\rm{exp }}}}}}\left({\lambda }_{{{{{{\rm{B}}}}}}}t\right)}{{\lambda }_{{{{{{\rm{A}}}}}}}-{\lambda }_{{{{{{\rm{B}}}}}}}},\hfill&{t} \, < \, {t}_{{{{{{\rm{max }}}}}}}\\ k\frac{{{{{{\rm{exp }}}}}}\left({\lambda }_{{{{{{\rm{A}}}}}}}{t}_{{{{{{\rm{max }}}}}}}\right)-{{{{{\rm{exp }}}}}}\left({\lambda }_{{{{{{\rm{B}}}}}}}{t}_{{{{{{\rm{max }}}}}}}\right)}{{\lambda }_{{{{{{\rm{A}}}}}}}-{\lambda }_{{{{{{\rm{B}}}}}}}}\times {{{{{\rm{exp }}}}}}\left({\lambda }_{{{{{{\rm{B}}}}}}}\cdot \left(t-{t}_{{{{{{\rm{max }}}}}}}\right)\right),&{t}\,\ge\, {t}_{{{{{{\rm{max }}}}}}}\end{array}\right.$$

These signals are proportional within identical factors to the time-dependent complex transverse magnetizations of **A** and **B** post-excitation, and they depend on the post-excitation time *t*, **A**’s initial concentration ${A}_{0}$ (in M), and the zero-order reaction rate constant $k$ (in M s^−1^). Here ${\lambda }_{{{{{{\rm{A}}}}}}/{{{{{\rm{B}}}}}}}=i{{{{{{\rm{\omega }}}}}}}_{{{{{{\rm{A}}}}}}/{{{{{\rm{B}}}}}}}-{R}_{2{{{{{\rm{A}}}}}}/{{{{{\rm{B}}}}}}}$, where ${{{{{{\rm{\omega }}}}}}}_{{{{{{\rm{A}}}}}}/{{{{{\rm{B}}}}}}}$ and ${R}_{2{{{{{\rm{A}}}}}}/{{{{{\rm{B}}}}}}}$ are the **A/B**-spin chemical shift (in rad/s) and transverse relaxation rates (in s^−1^) respectively, and *t*_max_ is a post-excitation time at which the chemical transformation is completed and all of **A** has been depleted. Supplementary Note 3 provides a derivation and detailed analysis of these equations, to which the presence of additional **B** at time *t* = 0 can also be added without excessive complications.

In order to add the effects of continuous flow onto Eqs. (3, 4) and account for positional changes in the magnetizations’ distributions, a spatial translation operator ${{{{{\mathscr{T}}}}}}({{z}})$ representing the action of the flow was included. The resulting post-excitation, flow-dependent, kinetic NMR signals for **A** and **B**—still in the absence of pulsed magnetic field gradients—are then given by integrals over all 0 *≤* *z ≤* *L*_z_ positions within the active coil volume, as:

5 $${S}_{{{{{{\rm{A}}}}}}}\left(t\right)\propto \left\{\begin{array}{cc}\int {S}_{{{{{{\rm{A}}}}}}}\left(z,t\right){dz}\propto \int {{{{{{\rm{M}}}}}}}_{+}^{{{{{{\rm{A}}}}}}}\left(z,t\right)\times {{{{{\rm{exp }}}}}}\left({\lambda }_{{{{{{\rm{A}}}}}}}t\right){dz},& t \, < \, \frac{{A}_{0}(z)}{k}\\ \hfill 0,& t\,\ge\, \frac{{A}_{0}(z)}{k}\end{array}\right.$$

6 $${S}_{{{{{{\rm{B}}}}}}}\left(t\right)\propto \left\{\begin{array}{cc}\int {M}_{+}^{{{{{{\rm{B}}}}}}}\left(z,t\right)\times {{{{{\rm{exp }}}}}}\left({\lambda }_{{{{{{\rm{B}}}}}}}t\right){dz}+\int {{{{{\mathscr{T}}}}}}\left\{\frac{k}{{\lambda }_{{{{{{\rm{A}}}}}}}-{\lambda }_{{{{{{\rm{B}}}}}}}}\right\}\times \left({{{{{\rm{exp }}}}}}\left({\lambda }_{{{{{{\rm{A}}}}}}}t\right)-{{{{{\rm{exp }}}}}}\left({\lambda }_{{{{{{\rm{B}}}}}}}t\right)\right){dz},\hfill&t < \frac{{A}_{0}\left(z\right)}{k}\\ \int {M}_{+}^{{{{{{\rm{B}}}}}}}\left(z,t\right)\times {{{{{\rm{exp }}}}}}\left({\lambda }_{{{{{{\rm{B}}}}}}}t\right){dz}+\int {{{{{\mathscr{T}}}}}}\left\{k\frac{{{{{{\rm{exp }}}}}}\left({\lambda }_{{{{{{\rm{A}}}}}}}{t}_{{{{{{\rm{max }}}}}}}(z)\right)-{{{{{\rm{exp }}}}}}\left({\lambda }_{{{{{{\rm{B}}}}}}}{t}_{{{{{{\rm{max }}}}}}}(z)\right)}{{\lambda }_{{{{{{\rm{A}}}}}}}-{\lambda }_{{{{{{\rm{B}}}}}}}}\right\}\times {{{{{\rm{exp }}}}}}\left({\lambda }_{{{{{{\rm{B}}}}}}}\left(t-{t}_{{{{{{\rm{max }}}}}}}(z)\right)\right){dz},&t\ge \frac{{A}_{0}\left(z\right)}{k}\end{array}\right.$$

${{{{{{\rm{M}}}}}}}_{+}^{{{{{{\rm{A}}}}}}}\left(z,t\right)$ and ${{{{{{\rm{M}}}}}}}_{+}^{{{{{{\rm{B}}}}}}}\left(z,t\right)$ represent respectively the **A** and **B** transverse magnetization amplitudes, which now have distinct kinetic and flow-based dependencies that take the form:

7 $${{{{{{\rm{M}}}}}}}_{+}^{{{{{{\rm{A}}}}}}}\left(z,t\right)\propto \left\{\begin{array}{cc}{{{{{\mathscr{T}}}}}}\left({A}_{0}\left(z\right)\right)-{kt}={{{{{\mathscr{T}}}}}}\left\{{{{{{{\rm{A}}}}}}}_{0}-\frac{k}{v}z\right\}-{kt},&t \; < \;\frac{{A}_{0}\left(z\right)}{k}\;{{{{{\rm{and}}}}}}\;t \; < \; \frac{{L}_{{{{{{\rm{z}}}}}}}-z}{v}\;{{{{{\rm{and}}}}}}\;t \; < \; \frac{z}{v}\\ \hfill 0,& \hskip-0.8pct\;\ge\; \frac{{A}_{0}\left(z\right)}{k}\;{{{{{\rm{or}}}}}}\;t \;\ge\; \frac{{L}_{{{{{{\rm{z}}}}}}}-z}{v}\;{{{{{\rm{or}}}}}}\;t\ge \frac{z}{v}\end{array}\right.$$

and,

8 $${{{{{{\rm{M}}}}}}}_{+}^{{{{{{\rm{B}}}}}}}\left(z,t\right)\propto \left\{\begin{array}{cc}{{{{{\mathscr{T}}}}}}\left({B}_{0}\left(z\right)\right)={{{{{\mathscr{T}}}}}}\left\{\frac{k}{v}z\right\},&t \; < \; \frac{{A}_{0}\left(z\right)}{k}{{{{{\rm{or}}}}}}\,t \;\ge\; \frac{{A}_{0}\left(z\right)}{k}\;{{{{{\rm{and}}}}}}\;t \; < \; \frac{{L}_{{{\rm{z}}}}-z}{v}\;{{{{{\rm{and}}}}}}\;t \; < \; \frac{z}{v}\\ \hfill 0,&\hskip-8.5pc t \;\ge\; \frac{{L}_{{{{{{\rm{z}}}}}}}-z}{v}\;{{{{{\rm{or}}}}}}\;t \;\ge\; \frac{z}{v}\end{array}\right.$$

**Fig. 5:**
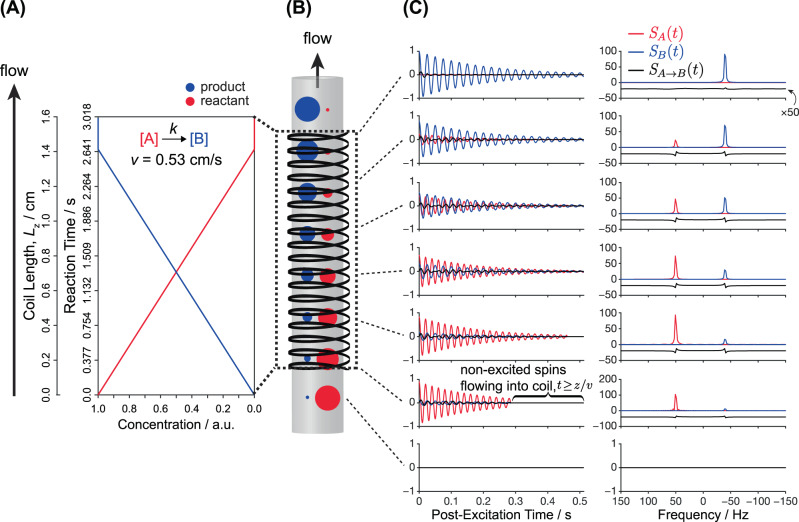
Flow-based spatiotemporal encoding of chemical kinetics. **A** Zero-order kinetic profile (*k* = 0.375 M s^−1^) assumed for an off-equilibrium reaction (**A**→**B**), plotted as a function of both reaction time and position for *v* = 0.53 cm/s and *L*_*z*_ = 1.6 cm discretized over 256 unique *z* positions. **B** Schematic representation of the spatiotemporally-encoded chemical kinetics along the *z* spatial coordinate of the NMR coil that results from plug-like flow. The relative spatially-dependent concentrations of **A** and **B** are schematized with the red and blue circles, respectively, with their corresponding simulated time-resolved 1D NMR FIDs and spectra shown in **C**, which were generated using Eqs. (5, 6) of the main text. Notice the different scaling factors of the colored and black traces.

Here ${{{{{\mathscr{T}}}}}}$ is the spatial translation operator taking as input ${A}_{0}(z)$ and ${B}_{0}(z)$, where ${A}_{0}(z)$ and ${B}_{0}(z)$ represent the spatially-dependent dynamic equilibrium magnetizations of **A** and **B**, respectively, at *t* = 0 immediately preceding excitation. ${{{{{\mathscr{T}}}}}}$ then translates these magnetizations from their initial positions $z$ at $t=0$, by an amount $\delta z={vt}$ as a function of the FID acquisition time. All other symbols possess similar definitions as given earlier. Notice that these Equations rely on $v$ as a unique, constant plug-flow rate in order to relate the evolution time *t* for a spin with its changing position within the NMR coil. Notice as well that in addition to the boundary condition accounting for the kinetic consumption of—a now spatially-dependent—*A*_0_(*z*) by the chemical reaction proceeding at a rate *k*, (*i.e*., by making the *t*_max_ of Eqs. (3, 4) equal to ${t}_{{{{{{\rm{max }}}}}}}$(*z*) = $\frac{{A}_{0}\left(z\right)}{k}$), a new set of flow-dependent boundary conditions are introduced in Eqs. (7, 8). These account for both excited magnetizations flowing out of the coil, which zeroes-out contributions for certain spins at times $t\ge \frac{{L}_{{{{{{\rm{z}}}}}}}-z}{v}$ as they have exited the coil’s field of view, as well as for the in-flow of non-excited (even if pre-polarized) magnetizations flowing into the coil at times $t\ge \frac{z}{v}$. Figure 5 clarifies further the time- and position-dependent magnetizations of **A** and **B** subjected to the flow- and kinetic-dependent boundary conditions given by Eqs. (7, 8), for *k* = 0.375 M s^−1^ and a linear flow rate of *v* = 0.53 cm/s. Figure 5A and B shows the time- and space-dependent magnetizations within a schematized NMR coil, illustrating the depletion and growth of the reactant (red) and product (blue) as a function of $z$.

As discussed in the Overall Principles section, the unique proportion of **A** and **B** for every position $z$ will correspond to definite moments along the reaction coordinate, reflecting in turn in the NMR correlations observed by 2D EPSI. The idealized (*e.g*., turbulence- and diffusion-free) time- and frequency-domain responses of these sites, are in Fig. 5C. Shown by red and blue traces are the NMR responses of **A** and **B**, respectively, that originate solely from the equilibrium magnetization established pre-excitation; shown in black are the NMR spectra of **B** signals that were generated post-excitation *via* the zero-order kinetics. Notice that the latter contain contributions from both sites, and a mixed-phase line shape reflective of the multiple *t*’ > 0 instants at which the **A**→**B** interconversion took place. These simulations also demonstrate that the contributions of these signals for the reaction conditions designed in the experiments above, are much smaller than the contributions present at $t=0$ pre-excitation, and hence they are not detectable; they would, however, be noticeable for faster reaction rate constants *k*. Note as well that the direction of the plug-like flow, assumed to proceed in a vertical, +*z* direction, causes a premature FID zeroing for both ${{{{{{\rm{M}}}}}}}_{+}^{{{{{{\rm{A}}}}}}}$ and ${{{{{{\rm{M}}}}}}}_{+}^{{{{{{\rm{B}}}}}}}$ located at $z$ positions nearest the bottom of the NMR coil at post-excitation times $t\ge \frac{z}{v}$ (lower traces in the figure). This manifests in the NMR spectra as a line broadening.

While these analytical simulations can be used to predict the kinetically-resolved 1D NMR spectra for distinct positions, a conventional 1D pulse-acquire experiment cannot deconvolve the individual spatially-dependent contributions to the **A** and **B** signals. Spatially-selective acquisitions employing field gradients are needed to unravel this information, as described in the next Paragraph.

### Real time gradient-driven decoding of the spatially encoded chemical kinetics

The spectrotemporal NMR method in Fig. 1 relies on EPSI to retrieve the aforementioned spatially-encoded kinetic information with chemical shift resolution. Modeling the effects of the EPSI time-dependent oscillating gradient readout with the zero-order chemical kinetics and the continuous plug-like flow can be accomplished by appending to the treatment in the preceding Paragraph, the relevant gradient-induced shifts that affect each spin as it changes position due to the flow, and as it changes chemical environment due to the chemical kinetics. In addition, the various boundary conditions detailed in the previous Paragraph need to be respected. Once again the NMR signal of the starting reactant **A** as a function of all these factors is easier to summarize, and is given by

9 $${S}_{{{{{{\rm{A}}}}}}}\left(t\right)\propto\left\{\begin{array}{cc}\int {{{{{{\rm{M}}}}}}}_{+}^{{{{{{\rm{A}}}}}}}\left(z,t\right)\times {{{{{\rm{exp }}}}}}\left({\lambda }_{{{{{{\rm{A}}}}}}}t\right)\times {{{{{\rm{exp }}}}}}\left(i\gamma {\int }_{0}^{t}{{{{{\rm{G}}}}}}\left({t}^{{\prime} }\right)\cdot z\left({t}^{{\prime} }\right){dt}^{\prime} \right){dz},& t \; < \; \frac{{A}_{0}(z)}{k}\\ \hfill 0,&\hskip0.5pc t\ge \frac{{A}_{0}\left(z\right)}{k},\end{array}\right.$$

where ${{{{{{\rm{M}}}}}}}_{+}^{{{{{{\rm{A}}}}}}}\left(z,t\right)$ is given by Eq. (7), $\gamma$ is the gyromagnetic ratio of the imaged spins in rad∙Hz/T, ${{{{{\rm{G}}}}}}\left({t}^{{\prime} }\right)$ is the time-dependent gradient amplitude in G/cm, and $z\left({t}^{{\prime} }\right)=z+{vt}^{\prime}$ is the time-dependent position of a given flowing spin at time $t^{\prime}$ that had an initial position $z$, with $0\le t^{\prime} \le t$. The new exponent in Eq. (9) highlights the fact that spins are undergoing motion while being imaged—a topic, which has been extensively discussed in the NMR and MRI literature^17,22–25^. As is well known from such studies, this gradient/flow combination can result in signal attenuations and/or in secular-like shifts of the signals. Supplementary Note 4 investigates this phenomenon in greater detail as it pertains to spectrotemporally-encoded NMR correlations, and demonstrates that in a pre-phased EPSI readout of the kind given in Eqs. (9–11), the spin evolution is compensated against the effects of plug-like flow. Therefore, the resulting NMR signals are devoid of flow-induced frequency shifts (even if they will be affected by flow-dependent broadening related to out-flowing of polarized spins and in-flowing of unpolarized/ones, *vide supra*).

The corresponding expression for **B** is composed in an analogous fashion as was carried out for **A**, in which each unique chemical shift term in Equation (S3.2) and Equation (S3.3) (Supplementary Note 3) is multiplied by its corresponding gradient-induced shift. After some algebra (see Supplementary Note 5 for the full derivation), the following piece-wise expression is obtained:

10 $${S}_{{{{{{\rm{B}}}}}}}\left(t\right) 	\propto {\int }_{Z}{M}_{+}^{{{{{{\rm{B}}}}}}}\left(z,t\right)\times {{{{{\rm{exp }}}}}}\left({\lambda }_{{{{{{\rm{B}}}}}}}t\right)\times {{{{{\rm{exp }}}}}}\left(i\gamma {\int }_{0}^{t}{{{{{\rm{G}}}}}}\left({t}^{{\prime} }\right)\cdot z\left({t}^{{\prime} }\right)d{t}^{{\prime} }\right){dz}\\ 	+{\int }_{Z}k\frac{{{{{{\rm{exp }}}}}}\left({\lambda }_{{{{{{\rm{A}}}}}}}t\right)-{{{{{\rm{exp }}}}}}\left({\lambda }_{{{{{{\rm{B}}}}}}}t\right)}{{\lambda }_{{{{{{\rm{A}}}}}}}-{\lambda }_{{{{{{\rm{B}}}}}}}}\\ 	\quad\times {{{{{\rm{exp }}}}}}\left(i\gamma {\int }_{0}^{t}{{{{{\rm{G}}}}}}\left({t}^{{\prime} }\right)\cdot \left(z+v{t}^{{\prime} }\right)d{t}^{{\prime} }\right){dz},t \; < \; \frac{{A}_{0}(z)}{k}$$

11 $${S}_{B}\left(t\right) 	\propto {\int }_{Z}{M}_{+}^{B}\left(z,t\right)\times {\exp }\left({\lambda }_{B}t\right)\times {\exp }\left(i\gamma {\int }_{0}^{t}G\left({t}^{{\prime} }\right)\cdot z\left({t}^{{\prime} }\right)d{t}^{{\prime} }\right){dz}\\ 	+{\int }_{Z}k\frac{{\exp }\left({\lambda }_{A}{t}_{{\max }}\right)-{\exp }\left({\lambda }_{B}{t}_{{\max }}\right)}{{\lambda }_{A}-{\lambda }_{B}}\times {\exp }\left({\lambda }_{B}\cdot \left(t-{t}_{{\max }}\right)\right)\\ 	\quad\times {\exp }\left(i\gamma {\int }_{0}^{t}G\left({t}^{{\prime} }\right)\cdot \left(z+{vt}^{\prime} \right)d{t}^{{\prime} }\right){dz},t\ge \frac{{A}_{0}(z)}{k}$$

where ${{{{{{\rm{M}}}}}}}_{+}^{{{{{{\rm{B}}}}}}}\left(z,t\right)$ is defined in Eq. (8), and the spatial integral extends over the relevant field-of-view. Equations (9–11) therefore describe all of the time-dependent spin- and space-based dynamics relevant to 2D spectrotemporal kinetic NMR correlations, and permit analytical calculations of the aforementioned experimental datasets. Supplementary Note 5 provides a more thorough analysis of the resulting theoretical EPSI line shapes described with Eqs. (9–11), by exploring three limiting cases in which: i) the spins evolve in the absence of the chemical kinetics (*i.e*., *k* = 0) under the effects of both field gradients and flow; ii) stopped-flow conditions are employed during the EPSI readout (*i.e*., *v* ≠ 0 pre-excitation and *v* = 0 post-excitation); iii) *v* > *k* leading to measurable flow-induced broadening under conditions of continuous flow during FID acquisition.

## Discussion

A method for monitoring off-equilibrium chemical transformations in real time by NMR was proposed and demonstrated. The method relies on the combined application of rapid-mixing, continuous flow, and single-scan spatial-spectral acquisition schemes. To test the method a custom syringe-driven flow apparatus allowing for the rapid mixing of selectively pre-polarized reagents was built, leading to a stable flow of reaction mixtures through a standard 5 mm commercial NMR probehead. This setup required no modifications of the remaining NMR hardware, as it relied on a mixing device designed to be loaded into a conventional 5 mm NMR tube and probe. The resulting continuous flow served to map the time coordinate of an off-equilibrium reaction onto the *z* spatial coordinate of the NMR coil. The flow-derived spatially-encoded kinetic NMR information was then retrieved using echo-planar spectroscopic imaging, which delivered the site-resolved resonances for the various reacting species from different *z* position in space and in a single scan. Experimental validation of the technique was performed by monitoring the enzymatically-catalyzed hydrolysis of an ester and the corresponding formation of ethanol, in which the methyl proton resonances of both were selectively targeted. The custom-built flow apparatus and associated hardware allowed for the collection of quality slice-selective and EPSI NMR data averaged over minutes, and the execution of experiments under different flow rates and spatial encoding conditions. The encoded kinetic NMR information could be extracted from these data and agreed remarkably well with both reported literature values and control experiments.

An assumption used in the design and analysis of the method’s results rested on the uniformity of the flow throughout the NMR detector; *i.e*., on the plug flow assumption. This simplifies the derivations yet in actuality, for the Reynolds numbers employed in our studies, the flow in the employed setup should have a parabolic profile. To quantify this flow velocity distribution across the transverse, radial axis, we relied on simulations performed using Ansys’s Fluent^®^, an advanced fluid dynamics calculation tool^26^. Supplementary Note 6 provides a description of the results arising for a variety of relevant flow profiles. These calculations entailed taking the formalism derived in the previous Section, and replacing the position independent spatial translation operator ${{{{{\mathscr{T}}}}}}$ (Eqs. (7, 8)) with radially-dependent ${{{{{\mathscr{T}}}}}}(y)$ operators accounting for the flow velocities predicted by Fluent^®^. The resulting simulations showed that minor deviations from the ideal peak intensities expected from a plug-flow-based analysis emerged upon considering more realistic flow profiles (Supplementary Figs. 11, 12).

In principle, this combined approach based on using flow to spatially encode chemical transformations followed by a joint NMR/MRI decoding of the spectral and spatial information can deliver a picture of the entire reaction—from an initial state consisting mostly of reactants to a final state consisting mostly of products—with ms time resolution and in a single scan. From an experimental standpoint, the most important limitation arising from this combined use of flow and spectroscopic imaging is given by sensitivity penalties and by the stability of the flow. The former arises as probing faster kinetic timescales will be associated with resolving smaller spatial elements, leading to decreased signals; the latter due to the fact that although the system is assumed to be spatially at a steady state, deviations in plug flow and/or turbulences will blur the kinetic information (Supplementary Note 2). In addition to these constraints another potential source of instability could arise from kinetic-induced changes in the chemical shifts, which although not witnessed in this study, could give rise to broadened resonanes in EPSI’s spectral dimension.

This study explored features of this method using an analytical model complemented by numerical simulations. These described the NMR spin dynamics for an off-equilibrium zero-order chemical reaction, while accounting for the EPSI acquisition under the combined effects of plug flow and chemical kinetics. This model showed that EPSI acquisitions of flowing off-equilibrium reactions contain signal contributions from two distinct sources: one from the reactants and products established pre-excitation, and the other from the products created over the course of FID acquisition post-excitation. For the experimental examples presented here, the former is the dominant contribution to the NMR line shape. The latter’s contributions, however, will become relevant when faster reaction rates are examined, as a greater proportion of the observed product signal gets created over the course of the FID acquisition. The mixed phase character of the resulting line shapes encodes additional information about the chemical kinetics. The theoretical model also showcased the conditions by which flow-based features arising from the inflow of unexcited, NMR-silent spins and the outflow of excited spins will manifest as spectral broadenings. These effects could be removed by reducing the imaged field-of-view, in combination with RF pulses having increased spatial excitation bandwidths: this would ensure the in-flow of excited spin magnetizations over the entire course of the FID acquisition. On-going work includes the targeting of faster reaction kinetics through the combined use of increasingly high-resolution spatial readouts, faster linear flow rates, and advanced modeling of the kinetics. A crucial requirement for achieving higher temporal reaction resolutions will be adequate sensitivity, which we are striving to achieve by incorporating hyperpolarization. Alternatively, the reliance on microfluidic components encompassing a high Reynolds number region for ensuring vigorous mixing immediately adjacent to a low-Re region of smooth flow within the coil observation region might provide the sensitivity/sample volume compromise needed for a more widespread application of this method^27–30^.

## Methods

### The mixer device

A mixer device was designed and constructed for continuous flow NMR with the capability to pre-polarize the nuclear spins of one of the reactants. The device was constructed to fit within a narrow-bore NMR magnet equipped with a commercial shim system. The mixer contains two inlet channels for the two reactants (Supplementary Fig. 1). One reactant passes through the middle inlet leading to the pre-polarization chamber (2.7 mL volume), which provides an increased residence time of nominally 3 s at a flow rate of 50 mL/min in order to polarize the spins. The second reactant is injected through the other inlet and mixes with the first reactant downstream of the pre-polarization chamber. Immediately below, the mixture flows through a leading tube (with 1/32 and 1/50 in. outer and inner diameters, respectively, IDEX Health & Science, Oak Harbor, WA) to the bottom of a standard 5 mm NMR tube inserted into the NMR probe. The reaction mixture then flows upward in the direction parallel to the main magnetic field through the NMR coil. The spent reaction solution eventually flows out from the return channel of the mixer and is collected in a waste container. The device was made from a cylinder of Delrin plastic with an outer diameter of 25 mm and a length of 75 mm (Online Metals, Seattle, WA). Channels of 1.6 mm width and depth were machined by computer numerical control (CNC) into a flat surface milled into the cylinder (letter A in Supplementary Fig. 1). The channels were sealed with a single gasket sheet made from nitrile rubber (McMaster-Carr, Elmhurst, IL). The cover (B) was attached with brass screws (4–40 unified thread standard). The NMR tube was attached below the mixer, using a nitrile O-ring between pieces (A) and (C), and a retaining ring permanently glued to the NMR tube between pieces (C) and (D). Mixing was improved with a cotton string inside a section of the leading tube (green tubing, 1/16 inch outer and 1/32 in. inner diameter, Fig. 1B).

### Pulse sequences and data processing

All NMR spectra were recorded using a triple resonance TXI probe installed in a 9.4 T NMR spectrometer (Bruker Biospin, Billerica, MA). The EPSI pulse sequence^13^ (Fig. 1D) used an excitation pulse that was frequency-selective to excite 400 Hz of the ^1^H methyl region (using a Pc9_4_90 shape) in τ_pw_ = 18.78 ms, which was experimentally optimized to give both uniform excitation in the frequency domain as well as in the spatial domain (Supplementary Fig. 2). An EPSI pre-phasing period of *T*_a_/2 = 0.25 or 0.5 ms was used along with associated readout times lasting *T*_a_ = 0.5 or 1 ms, with all gradients having an amplitude of $\left|{G}_{{{{{{\rm{z}}}}}}}\right|$ = 20.5 G/cm. The pair of bipolar gradients was looped *N*_EPSI_ times, in which 2*N*_EPSI_ gradient echoes were recorded. All EPSI NMR spectra were generated by rearranging the 1D gradient echo trains into 2D matrices, in which only the odd echoes were used for processing. Processing included Gaussian multiplication and zero-filling in both dimensions, followed by 2D Fourier transformation with respect to *k*_z_ and 2*N*_EPSI_*T*_a_ along each row and column, respectively. All data are presented in magnitude mode. A spatial resolution of ∆*z* = 1/γ*G*_z_*T*_a_ = 115 μm was used in the EPSI measurements unless stated otherwise. The spectral width is given by twice the spacings between adjacent gradient echoes (*i.e*., *DW* = 1 or 2 ms and *SW* = 1000 or 500 Hz, respectively), which gives a spectral resolution of 10 or 5 Hz/pt. A G4 Gauss-Cascade pulse^14^ with a bandwidth of 15 kHz was used for slice-selective excitation experiments in the presence of a *z* gradient with amplitude $|{G}_{z}|$ = 17.62 G/cm, resulting in a slice thickness of *ca*. 2 mm. A total of 8 slices within the sensitive region of the NMR coil were measured successively using frequency offsets covering a total of 105 kHz with a step size of 15 kHz. A water suppression sequence consisting of an EBURP 2.1 shaped π/2 pulse followed by randomized pulsed field gradients *G*_x_, *G*_y_, and *G*_z_, applied before the excitation pulse. Data acquisition lasted 1 s with a total of 6400 complex points acquired in a single slice. A simple spectral normalization procedure was carried out for all EPSI NMR datasets. This procedure corrected for discrepancies between ^1^H peak intensities in the EPSI ^1^H NMR spectra and conventional 1D ^1^H NMR spectra that were collected on a stationary (*i.e*., non-flowing) mixture of BAEE and ethanol. In this case, the methyl peak corresponding to the ethanol had a larger intensity than that of BAEE in the 1D ^1^H NMR spectrum, whereas the reverse was the case in the EPSI NMR spectrum. Since the total concentration of the reactant and the product remains constant for a given position under conditions of continuous flow, the peak integrals of both were normalized before kinetic fitting according to: ${\hat{S}}_{{{{{{\rm{R}}}}}}}={S}_{{{{{{\rm{R}}}}}}}/\left({S}_{{{{{{\rm{R}}}}}}}+{S}_{{{{{{\rm{P}}}}}}}\right)$ and ${\hat{S}}_{{{{{{\rm{P}}}}}}}={S}_{{{{{{\rm{P}}}}}}}/\left({S}_{{{{{{\rm{R}}}}}}}+{S}_{{{{{{\rm{P}}}}}}}\right)$, whereby the R and P subscripts refer to the reactant and product integrals respectively. Furthermore, due to the characteristic 1D spatial coil profile recorded in EPSI NMR spectra whereby the signal intensity falls off at the edges, only signals originating from within the ±0.7 cm region from the center of the coil were considered for kinetic analysis (Supplementary Fig. 3).

### Sample preparation

The substrate solution was prepared by dissolving *N*-α-benzoyl arginine ethyl ester (BAEE, TCI Chemicals, Tokyo, Japan) in 50 mM pH 7.6 phosphate buffer. The enzyme trypsin (AMERSCO, Road Solon, OH) was dissolved in the same buffer at a concentration of 7.5 or 25 µM (depending on the flow rate). 10% D_2_O was added to the substrate solution for frequency locking. The substrate and enzyme solutions were injected into the mixer device in a 1:1 volume ratio with the use of a syringe pump (Fusion 200 Touch, Chemyx, Stafford, TX). Notice that although in the ensuing sample the enzyme-substrate system may be in equilibrium, the EPSI experiment measures a unidirectional Enzyme+Substrate ⇌ Enzyme-Substrate → Product process that proceeds towards completion as a function of reaction/flow time.

## Supplementary information

Supplementary Information

## Data Availability

The raw data sets and the spectrotemporal images generated and analyzed during the current study are available from the corresponding authors on request. Also available upon request are machine drawings of the mechanical parts made for this work, simulated data sets, etc.

## Code availability

The MATLAB-based code used for simulating experimental datasets is available from the corresponding authors on request. So are the NMR pulse sequences used in this study.
