# Supplementary material for: Time- and site-resolved kinetic NMR for real-time monitoring of off-equilibrium reactions by 2D spectrotemporal correlations
Source: Nat Commun. 2022 Feb 11;13:833. doi: 10.1038/s41467-022-28304-w (PMC8837778; doi:10.1038/s41467-022-28304-w)
Supplement: Supplementary file 1 — Supplementary Information [file 41467_2022_28304_MOESM1_ESM.pdf]

Supplementary Information

Time- and Site-Resolved Kinetic NMR for Real-Time Monitoring of Off-Equilibrium Reactions by 2D Spectrotemporal Correlations

Michael J. Jaroszewicz,[1] Mengxiao Liu,[2] Jihyun Kim,[2] Guannan Zhang,[2] Yaewon Kim,[2] Christian Hilty[2,*], Lucio Frydman[1,*]

Affiliations

1 Department of Chemical and Biological Physics, Weizmann Institute of Science, 7610001 Rehovot, Israel

2 Chemistry Department, Texas A&M University, 3255 TAMU, College Station, TX, USA

Supplementary Note 1. **Additional experimental details concerning the mixer device, the methyl-selective excitation scheme, and the kinetic analysis.**

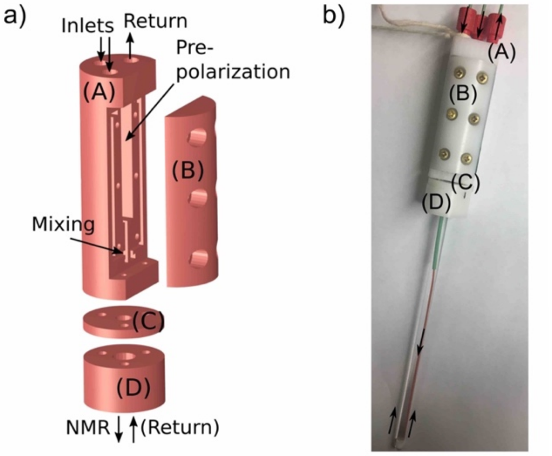

Supplementary Figure 1. **Schematics of the custom-built mixer device.** (a) Expanded view of the device for rapid mixing flow-NMR spectroscopy showing the channel substrate (A), cover plate (B), as well as the NMR tube adapter consisting of the sealing plate (C) and the tube retainer (D). (b) The fabricated device integrated with a standard 5 mm NMR tube and fluid connectors.

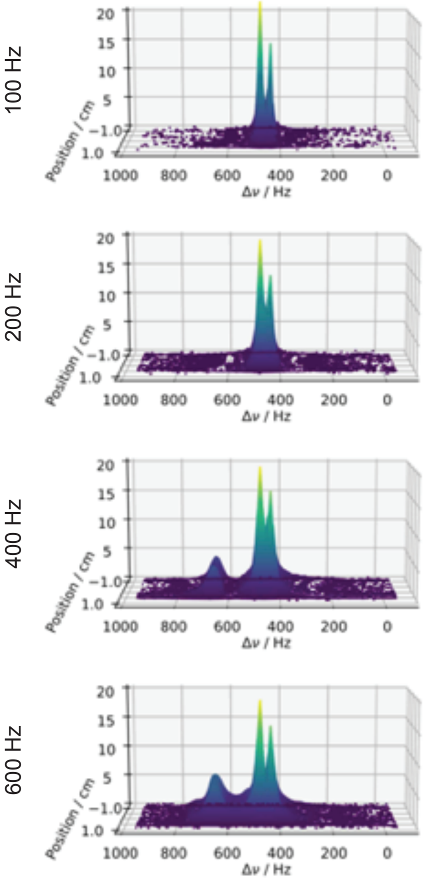

Supplementary Figure 2. **Experimental optimization of the ^{1}H methyl selective excitation pulse.** Experimental ^{1}H EPSI NMR spectra showing the spectral dimension recorded at different spectral excitation bandwidths centered at the methyl region. A static (*i.e.*, non-flowing) and non-reacting mixture of BAEE and ethanol was used.

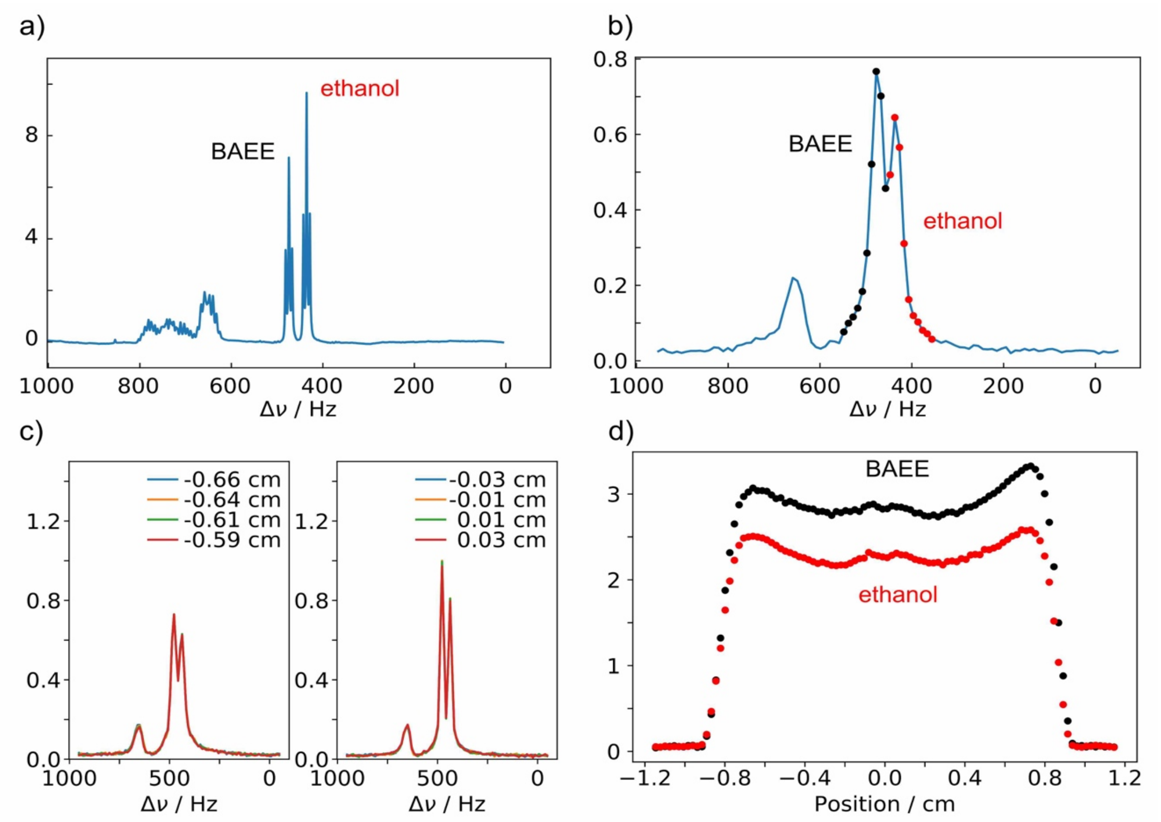

Supplementary Figure 3. **Integrating the ^{1}H EPSI NMR reactant and product peaks for kinetic analysis.** a) 1D ^{1}H NMR spectrum of a mixture of BAEE and ethanol. The integral ratio of ethanol over BAEE, α, is larger than 1. b) Integration regions, indicated by the circles, for the reactant (black) and product (red). c) Spectral cross sections taken from two distinct regions along the field-of-view: from the edge of the coil (left) and in the middle of the coil (right). d) The integrals of BAEE (I_R) and ethanol (I_P) are plotted against position, which were further corrected to match the relative intensities measured in (a). Prior to the kinetic analysis the integrals IR and IP for each position were normalized according to $\hat{S}_R = S_R/(S_R + S_P)$ and $\hat{S}_P = S_P/(S_R + S_P)$. Only the signals contained with ± 0.7 cm of the center of the coil were used in the kinetic analysis, due inefficient excitation outside this region.

Supplementary Note 2. **On experimental accuracy: The effects of flow rate, imaging spatial resolution, and temporal resolution, on the signal-to-noise ratio.**

The effects of spatial resolution on the signal-to-noise ratio (SNR) of spectrotemporal 2D NMR correlations can be inferred from the usual relationships between SNR and resolution in MRI.[1] Assuming that the SNR of a conventional NMR experiment on the sample is given by

$$SNR_{\mathrm{conv}} \propto \frac{M_0}{n_{\mathrm{rms}}} \qquad \text{(S2.1)}$$

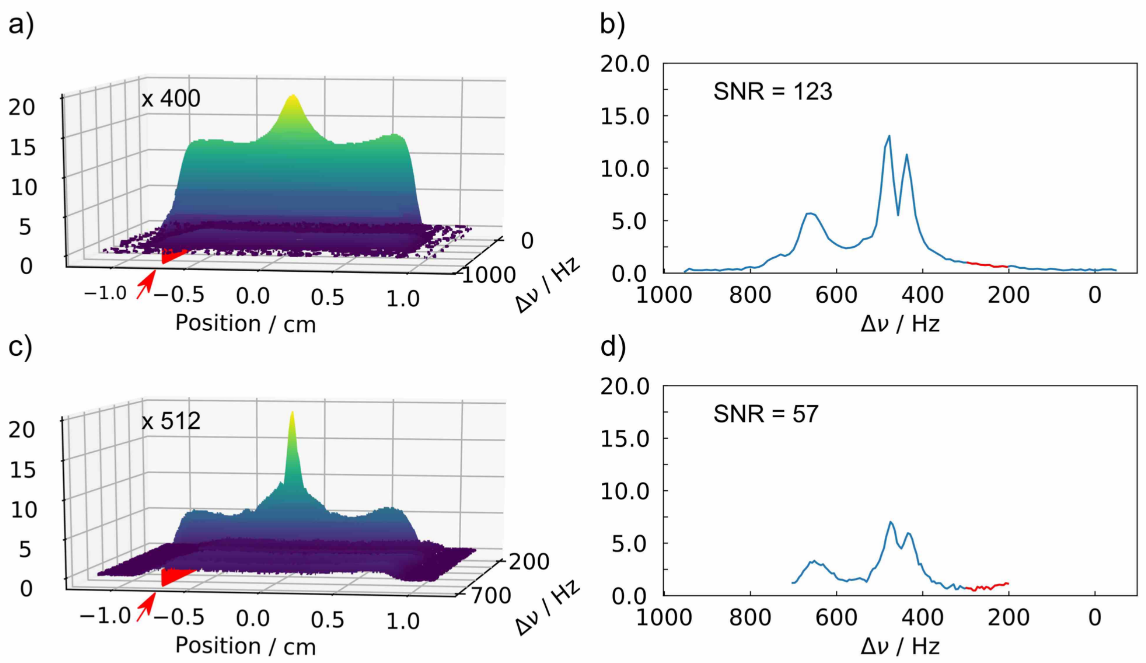

Supplementary Figure 4. SNR measurements for ^{1}H EPSI NMR spectra of non-flowing and non-reacting mixtures at different imaging spatial resolutions. ^{1}H EPSI NMR spectra comparing the SNR at different spatial resolutions (a-b, Δz = 230 µm and c-d, Δz = 115 µm) using a non-flowing and non-reacting sample mixture consisting of BAEE and ethanol. All other experimental gradient conditions are identical to those described in the main text. The red arrow in the 3D plots show the region (z = 0.685 cm) where the SNR measurements are performed along the spatial dimension, whereas the red regions in the corresponding spectral dimension to the right indicate the noise regions that were considered. The SNR values are reported in the figure as well as the number of scans acquired in each case.

where M_0 and n_{rms} denote the targeted site's magnetization and the root-mean-square of the noise, then, for a given spatial resolution Δz of the EPSI experiment, SNR will decrease as:[2]

$$SNR_{\text{EPSI}} = SNR_{\text{conv}} \frac{\Delta z}{L} \qquad \text{(S2.2)}$$

where L is the overall sample length along z, and it is assumed that no diffusion-induced losses occur. This SNR dependence on spatial resolution is illustrated experimentally in Supplementary Figure 4, which shows that for a non-reacting and non-flowing mixture of BAEE and ethanol, increasing the spatial resolution from *ca*. 230 µm to *ca*. 115 µm reduces the SNR by a factor of *ca*. 2. This experimental demonstration is also confirmed by simulations (Supplementary Figure 5), conducted to investigate the relative contributions of spatial resolution and flow rate on the SNR, under the plug-flow approximation. In such a case, increasing flow rates do not result in SNR reductions. The SNR values for Supplementary Figures 5a and 5e, which were simulated with identical gradient readout conditions (Δz = 230 µm for both), but at two distinct non-zero flow

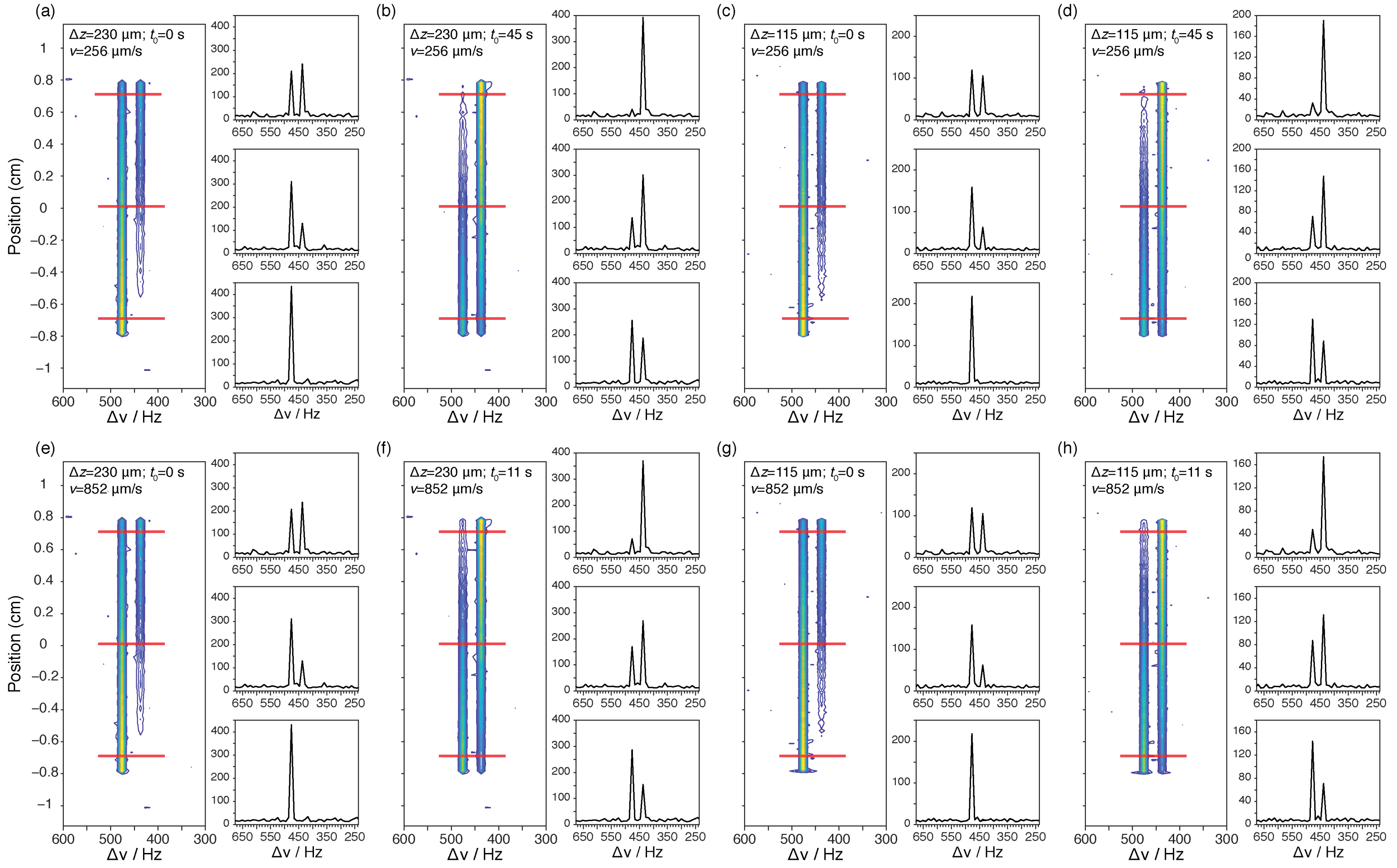

Supplementary Figure 5. Numerical simulations on the effects of the imaging spatial resolution Δ*z*, dead time t_0, and flow rate *v*, on the SNR of 2D spectrotemporal correlations. Simulations assumed an off-equilibrium binary chemical reaction between BAEE and trypsin (monitoring the methyl regions as a function of time/position) undergoing plug flow. All the parameters, except for the concentrations assumed, are indicated in each figure. The parameters used in these simulations mirror their experimental counterparts as closely as possible. The horizontal red bars indicate the positions from which the 1D time-resolved NMR spectra are extracted, which are displayed to the right of their corresponding 2D contour**.** Besides the indicated parameters, the initial concentration of the enzyme trypsin was increased from 7.5 μM to 25 μM for the faster flow rate (*v* = 852 μm/s, smaller dead time) in order to accurately capture the zero-order rate kinetics in the sensitive region of the NMR coil (matching the conditions of the experiment). White normally-distributed Gaussian noise was added to each dataset. The corresponding SNR values are recorded in Supplementary Table 1. All other simulation details are as discussed in the Methods section of the main text.

rates (v = 256 and 852 μm/s, respectively) are similar for each of the corresponding spectral cross sections (see Supplementary Table 1). Furthermore, the SNR values measured for Supplementary Figures 5c and 5g, which were simulated to deliver higher spatial imaging resolution (Δz = 115 μm for both) are also similar for each of the corresponding spectral cross sections. Therefore, these simulations show that *–as long as there is no turbulence–* increasing the flow rate whilst holding all other acquisition parameters fixed, does not result in significant changes to the SNR. Something similar should apply in the case of laminar flow; *i.e.,* as long as the rate of longitudinal flow remains constant and the oscillating gradients succeed in performing an ideal echo train.

Better understanding of the flow rate effects on the SNR, however, is relevant: experiments show that, contrast to the simulations above, flow does reduce SNR. Modelling this is challenging as one must be able to accurately account for the effects of turbulent flow –something our single-axis gradient system is ill-equipped for measuring. In the absence of this, experimental tests were conducted at different flow rates to examine the sensitivity losses. The SNR was seen decreasing in a manner that was commensurate with increasing flow rates (Supplementary Figure 6): for instance, under otherwise similar experimental conditions, increasing the flow rate by ≈3.3× decreased the SNR by a factor of *ca*. 2 (Supplementary Figure 6a *vs*. Supplementary Figure 6c). Simulations assuming random turbulences (not shown) also led to the introduction of noise in the spectral-containing regions.

It is enlightening to consider how the kinetic time resolution Δt_{R} will affect the available SNR. Assuming that the off-equilibrium reaction mixture is flowing with a uniform velocity v, the corresponding temporal resolution will be $\Delta t_{\mathrm{R}} = \Delta z/v$. Hence, following Eq. (S2.2), the experiment's sensitivity will be related to the latter as

$$SNR_{\mathrm{EPSI}} = \frac{M_0}{L \cdot n_{\mathrm{rms}}} v \cdot \Delta t_{\mathrm{R}} \quad \text{(S2.3)}$$

Since in general $v \cdot \Delta t_{\mathrm{R}} << L,$ the SNR of these experiments will therefore be reduced *vs* that of a conventional counterpart; this is expected for an imaging-based acquisition. Further, for a fixed flow velocity, an improvement in the kinetic time resolution *–i.e.,* a decrease in Δt_{R}– will be associated with a concomitant reduction in SNR. Under ideal conditions one might also increase

the kinetic time resolution without SNR penalties by increasing v, as a faster flow will reveal more details about the kinetics without changing the spatial resolution. As shown in Supplementary Figure 6, however, SNR decreases in our system with increasing v as a result of flow non-idealities.

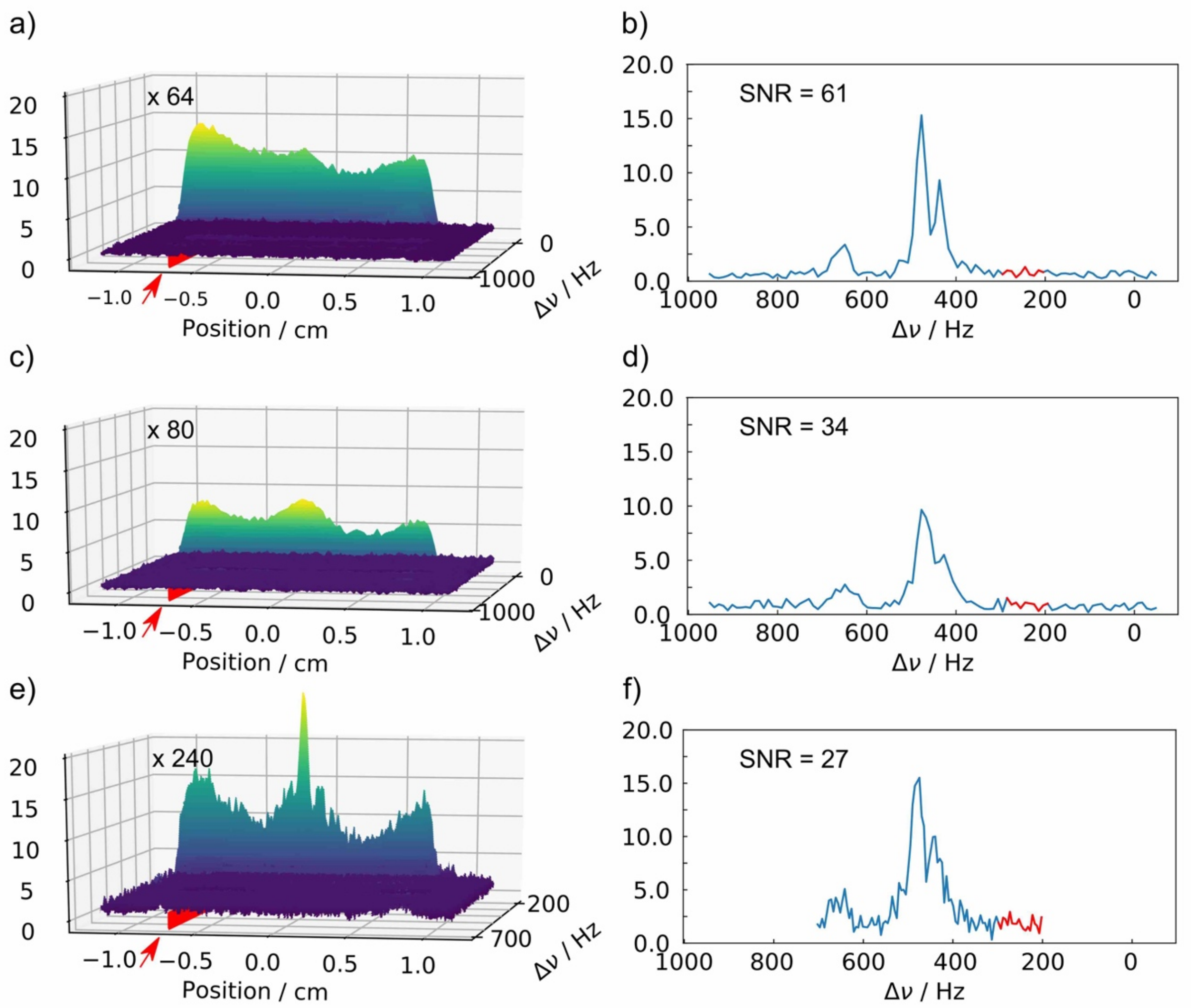

Supplementary Figure 6. SNR measurements for ^{1}H EPSI NMR spectra of non-reacting mixtures at different imaging spatial resolutions and flow rates. SNR comparisons for ^{1}H EPSI NMR spectra collected under various flow and gradient-readout conditions for the same non-reacting BAEE and ethanol mixture used in Supplementary Figure 4. The 3D view of the EPSI NMR spectra are shown in the left column and their corresponding spectral slices taken at the indicated spatial positions (z = 0.685 cm as indicated by the red arrows) are displayed to the right. The red regions in the ^{1}H spectra in the right column indicate the noise regions (which were all the same) used for SNR measurements. The spatial resolution for the datasets presented in (a,b) and (c,d) is Δz = 230 μm whereas for (e,f) Δz = 115 μm. These were again achieved by appropriately adjusting the length of the readout gradient as described in the **Methods section**. The flow rate is v = 0.3 mL/min and v = 1 mL/min for (a-b) and (c-f), respectively. The SNR values are reported in the figure, as well as the number of scans used in each case (left column). For experiments conducted with the faster flow rate, the initial concentration of the enzyme was increased to $[E]_0$ = 25 μm.

Finally, Supplementary Table 2 complements these experimental and numerical sensitivity

considerations, by presenting related features for experimental results shown in the manuscript's main text.

Supplementary Table 1: SNR values measured in the numeric 1D time-resolved NMR simulations (Supplementary Figure 5**)**

**Position (cm) \ Supplementary Figure 5**	**(a)**	**(b)**	**(c)**	**(d)**	**(e)**	**(f)**	**(g)**	**(h)**
**0.7 (top)**	30.1	52.0	12.7	3.0	25.8	7.9	12.8	4.8
**0 (middle)**	42.8	41.0	19.1	8.2	42.9	22.5	19.2	10.3
**−0.7 (bottom)**	53.6	31.1	32.8	19.7	53.1	34.9	32.4	21.9

Supplementary Table 2: Selected comparisons between the 2D spectrotemporal and optimized slice-selective experiments in Figures 2, 4 (respectively).

	**EPSI**	**1D Slice-selective**
**SNR**	55, 19	30, 32
**Acquisition time (minutes)**	5.3, 20	1, 1
**Temporal resolution (ms)**	580, 86	5020, 744
**Spectral resolution (Hz/pt)**	10, 5	0.94, 0.94

Supplementary Note 3. **Theoretical 1D NMR Spectra of Off-Equilibrium Irreversible Zero-Order Chemical Kinetics in the Absence of Flow and Field Gradients.**

This paragraph provides a description for the NMR response of a reacting off-equilibrium chemical reaction characterized by a zero-order rate law (*i.e.*, $A \xrightarrow{k} B$), leading to the corresponding theoretical 1D NMR line shapes. The resulting model serves as a convenient starting point for

elucidating the combined effects of plug-like flow and externally applied oscillating magnetic field gradients, which is discussed in the main text and elaborated upon in Supplementary Note 4. The reactant and product species are taken to be uniquely characterized by a single chemical environment. Each one is described by an isotropic chemical shift, whilst ignoring the effects of all other internal magnetic interactions. It is assumed here that initially only the reactant species **A** is present, and that its pre-polarized longitudinal equilibrium magnetization $M_{\mathrm{z}}^{\mathrm{A}}(t=0)$ is proportional to its concentration $[A(t=0)] = A_0$ for all positions z within the NMR coil. Then, at $t = 0$, the **A** spin magnetization is instantaneously and homogeneously (*i.e.*, for all positions) excited to the transverse plane and the chemical transformation is simultaneously initiated, which results in the progressive formation of the product species **B** – at the expense of the depletion of **A** – over the course of the FID acquisition. Ignoring the effects of relaxation for the moment, the change in the complex transverse magnetization for both **A** and **B** (denoted as $\mathrm{M}_{+}^{\mathrm{A}}(t)$ and $\mathrm{M}_{+}^{\mathrm{B}}(t)$, respectively) post excitation depends on their zero-order kinetics, in which the former and the latter decrease and increase linearly with time, respectively. The reaction progresses up to a point when all of **A** is expended (Supplementary Figure 7a). At this point in time, which we denote as t_{max}, the complex transverse magnetization of **A** (as well as its concentration) is 0, and remain so for all $t \geq t_{\mathrm{max}}$. Concomitantly, the absolute magnitude of the transverse magnetization for **B** becomes equal to A_0 for all $t \geq t_{\mathrm{max}}$. The **A** spin NMR signal is relatively straightforward to model. It is the product between its time-dependent complex transverse magnetization and its chemical shift evolution (accounting for the effects of transverse relaxation T_2), as given by:

$$S_{\mathrm{A}}(t) \propto \begin{cases} M_{+}^{\mathrm{A}}(t) \times \exp(\lambda_{\mathrm{A}} t) = (A_0 - kt) \times \exp(\lambda_{\mathrm{A}} t), & t < t_{\mathrm{max}} \\ 0, & t \geq t_{\mathrm{max}} \end{cases} \quad \text{(S3.1)}$$

where A_0 is the initial concentration of **A** in units of M (mol/L), k is the zero-order reaction rate in units of M s^{-1} and $\lambda_{\mathrm{A}} = i\omega_{\mathrm{A}} - R_{2\mathrm{A}}$, where ω_{A} and $R_{2\mathrm{A}}$ are the **A**-spin chemical shift in rad/s and transverse relaxation rate in s^{-1}, respectively. This is Equation (3) in the main text.

Describing the time evolution of **B** needs to account that the entirety of **B** progressively forms from **A** over the course of the FID acquisition. These spins will therefore spend some finite amount of time $0 \leq t < t'$ evolving as **A** under the effects of ω_{A}, $R_{2\mathrm{A}}$, and the **A**-spin chemical kinetics, accumulating a complex exponent proportional to $\lambda_{\mathrm{A}} t$. Then, at some time point $t = t'$, **B** is instantaneously formed with probability $P_{\mathrm{A}\rightarrow\mathrm{B}}(t') = k$. By then the spin packet has accrued

an exponential factor equal to $\lambda_{\mathrm{A}} t'$; subsequently it evolves under the effects of the **B**-spin characteristics (ω_{B},$R_{2\mathrm{B}}$) for $t' \leq t < t_{\max}$. This unitary transformation and subsequent transfer of phase information from **A** → **B** continues until all of **A** has reacted (*i.e.*, when $t = t_{\max}$), at which point the **B** transverse complex magnetization – which is itself comprised of discrete t'-dependent **A**-spin phase components – evolves in unison at the chemical shift of **B** for all $t \geq t_{\max}$. Mathematically, the **B** signal can thus be represented in terms of these two distinct time-dependent contributions as:

$$S_{\mathrm{B}}(t < t_{\max}) \propto \int_0^t P_{\mathrm{A}\to\mathrm{B}}(t') \times \exp(\lambda_{\mathrm{A}} t') \times \exp\big(\lambda_{\mathrm{B}} \cdot (t - t')\big) dt', \qquad \text{(S3.2)}$$

and,

$$S_{\mathrm{B}}(t \geq t_{\max}) \propto \left\{\int_0^{t_{\max}} P_{\mathrm{A}\to\mathrm{B}}(t') \times \exp(\lambda_{\mathrm{A}} t') \times \exp\big(\lambda_{\mathrm{B}} \cdot (t_{\max} - t')\big) dt'\right\} \times \exp\big(\lambda_{\mathrm{B}} \cdot (t - t_{\max})\big), \qquad \text{(S3.3)}$$

where $P_{\mathrm{A}\to\mathrm{B}}(t') = k$ is the probability for the reaction to occur at t' and $\lambda_{\mathrm{B}} = i\omega_{\mathrm{B}} - R_{2\mathrm{B}}$, which has the same units as the λ_{A} defined above. These integrals readily evaluate to the following expressions to give the signal of **B** for all post-excitation times t:

$$S_{\mathrm{B}}(t < t_{\max}) \propto k \exp(\lambda_{\mathrm{B}} t) \int_0^t \exp\big((\lambda_{\mathrm{A}} - \lambda_{\mathrm{B}}) t'\big)\, dt'$$

$$S_{\mathrm{B}}(t < t_{\max}) \propto \left(\frac{k}{\lambda_{\mathrm{A}} - \lambda_{\mathrm{B}}}\right) \times \exp(\lambda_{\mathrm{B}} t) \times \left\{\exp\big((\lambda_{\mathrm{A}} - \lambda_{\mathrm{B}}) t'\big)\right\}\Big|_{t'=0}^{t'=t}$$

$$S_{\mathrm{B}}(t < t_{\max}) \propto \left(\frac{k}{\lambda_{\mathrm{A}} - \lambda_{\mathrm{B}}}\right) \times \left(\exp(\lambda_{\mathrm{A}} t) - \exp(\lambda_{\mathrm{B}} t)\right) \qquad \text{(S3.4)}$$

and,

$$S_{\mathrm{B}}(t \geq t_{\max}) \propto k \exp(\lambda_{\mathrm{B}} t_{\max}) \times \left\{\int_0^{t_{\max}} \exp\big((\lambda_{\mathrm{A}} - \lambda_{\mathrm{B}}) t'\big) dt'\right\} \times \exp\big(\lambda_{\mathrm{B}} \cdot (t - t_{\max})\big) \qquad \text{(S3.5)}$$

$$S_{\mathrm{B}}(t \ge t_{\mathrm{max}}) \propto \left(\frac{k}{\lambda_{\mathrm{A}} - \lambda_{\mathrm{B}}}\right) \times \exp(\lambda_{\mathrm{B}} t_{\mathrm{max}}) \times \left\{\exp\left((\lambda_{\mathrm{A}} - \lambda_{\mathrm{B}})t'\right)\right\}\Big|_{t'=0}^{t'=t_{\mathrm{max}}} \times \exp\left(\lambda_{\mathrm{B}} \cdot (t - t_{\mathrm{max}})\right)$$

$$S_{\mathrm{B}}(t \ge t_{\mathrm{max}}) \propto \left(\frac{k}{\lambda_{\mathrm{A}} - \lambda_{\mathrm{B}}}\right) \times \left(\exp(\lambda_{\mathrm{A}} t_{\mathrm{max}}) - \exp(\lambda_{\mathrm{B}} t_{\mathrm{max}})\right) \times \exp\left(\lambda_{\mathrm{B}} \cdot (t - t_{\mathrm{max}})\right)$$

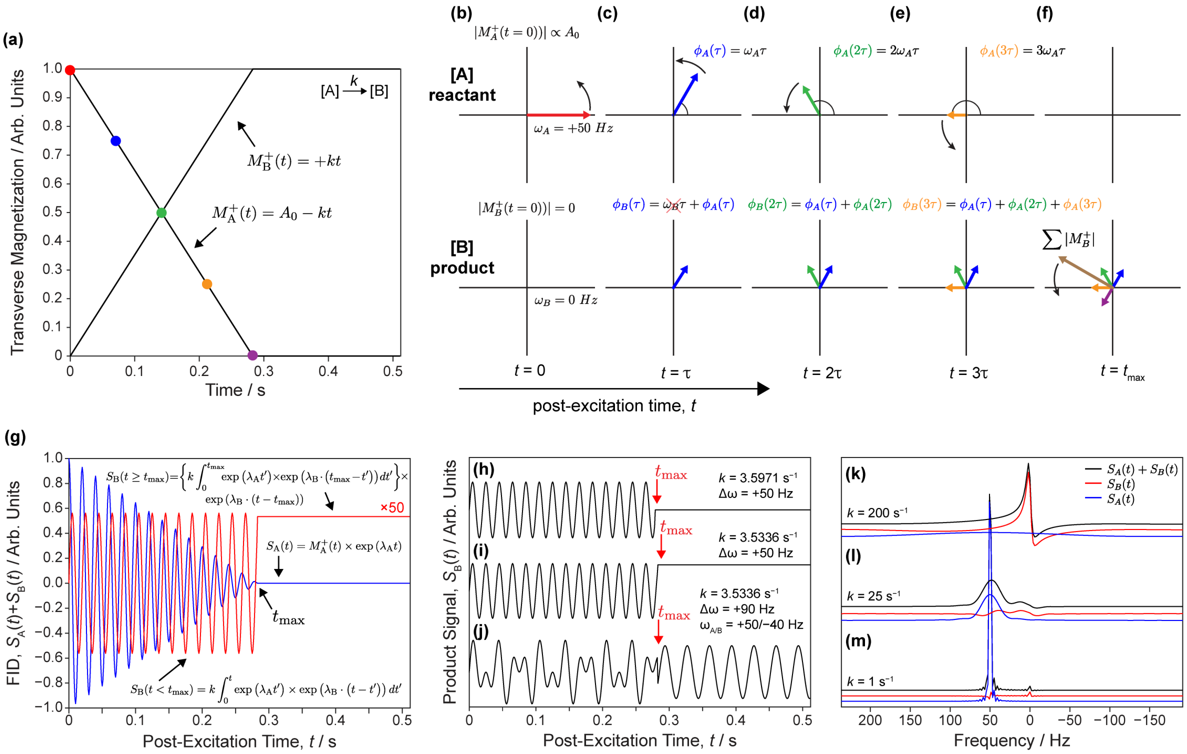

Supplementary Figure 7. Time-dependent transverse magnetization dynamics of A and B evolving from zero-order chemical kinetics occurring over the course of the FID acquisition. (a) The coloured points indicate distinct time points throughout the reaction process and correspond with the vector model presented in (b)-(f), which schematically illustrates the dynamics of **A** and **B** in a rotating reference frame that is resonant with the chemical shift of **B**. In this case, **A** oscillates with a +50 Hz frequency, whereas the magnetization vector elements of **B**, which are continuously formed throughout the reaction process, remain stationary. (g) FID evolution of **A** (blue trace) and **B** (red trace, × 50) for $\omega_{\mathrm{A}} = +50$ Hz, $\omega_{\mathrm{B}} = 0$ Hz, and $k = 3.5336\ \mathrm{s}^{-1}$. (h)-(j) Illustrations of the **B** signal evolution for different exchange rates and chemical shift separations (values indicated in the figure). (k)-(m) 1D NMR line shapes simulated at different exchange rates (values indicated in the figure) for $\omega_{\mathrm{A}} = +50$ Hz and $\omega_{\mathrm{B}} = 0$ Hz.

Combining Equations (S3.) and (S3.) gives the piece-wise expression for the evolution of the **B**-spin NMR signal:

$$S_{\mathrm{B}}(t) \propto \begin{cases} \left(\dfrac{k}{\lambda_{\mathrm{A}} - \lambda_{\mathrm{B}}}\right) \times (\exp(\lambda_{\mathrm{A}} t) - \exp(\lambda_{\mathrm{B}} t)), & t < t_{\max} \\ \left(k \dfrac{(\exp(\lambda_{\mathrm{A}} t_{\max}) - \exp(\lambda_{\mathrm{B}} t_{\max}))}{\lambda_{\mathrm{A}} - \lambda_{\mathrm{B}}}\right) \times \exp\big(\lambda_{\mathrm{B}} \cdot (t - t_{\max})\big), & t \geq t_{\max}. \end{cases} \quad \text{(S3.6)}$$

This is Equation (4) in the main text. Contributions to the **B**-spin NMR signal arising from excited equilibrium magnetization present at $t = 0$ are straight-forward to account for, and are described by a complex exponential akin to that in Equation (S3.1), evolving at λ_{B} and possessing a pre-exponential magnetization value that is time independent (*i.e.*, independent of the chemical kinetics).

The spin dynamics described by Equations (S3.1) and (S3.6) can be represented with a simplistic vector model in which the evolution of the bulk transverse magnetization for both the reactant and the product are independently examined in the same rotating reference frame (Supplementary Figures 7b-7f). This description can be further simplified by assuming that the transmitter frequency is resonant with ω_{B}, and therefore the chemical shift evolution of the product NMR signal is conveniently equal to zero. The plots in Supplementary Figures 7b-7f display snapshots of the evolution of the magnetization for **A** and **B** (top and bottom, respectively) at regularly spaced τ intervals throughout the reaction/acquisition, which are designated by the distinct colours that correspond to the coloured sampling points along the reaction coordinate as indicated in Supplementary Figure 7a. Initially, at $t = 0$ the **A**-spin magnetization is excited along the $+x$ axis of the rotating frame (Supplementary Figure 7b, red vector), which in this example begins to evolve with a +50 Hz chemical shift – there is no measurable **B** magnetization at $t = 0$. At an instant in time $t = \tau$ post excitation, the **A** spin transverse magnetization acquires a phase proportional to $\omega_{\mathrm{A}}\tau$, whilst simultaneously giving rise to a discrete transverse **B** magnetization vector that possesses the same **A**-spin phase and whose magnitude is proportional to $k\tau$ (Supplementary Figure 7c). Immediately after this instant of time, the **A**-spin magnetization continues to precess at +50 Hz and decays according to the zero-order kinetics (this is schematically indicated by a shortening of the **A** vector magnitude as described by Equation (S3.1)), whilst the component of **B** created precisely at $t = \tau$ remains stationary with a discrete phase given solely by $\phi_{\mathrm{A}}(\tau)$ (*i.e.*, since $\omega_{\mathrm{B}} = 0$ Hz and the newly formed **B** magnetization element does not accumulate any additional phase when the transmitter is on resonance with the product

peak), and is affected only by T_2 relaxation. At a later time, $t = 2\tau$, another discrete packet of **B** magnetization is formed that again has a magnitude proportional to $k\tau$, but at this moment possesses a phase equal to $2\omega_A\tau$ that continues to remain stationary for all t (Supplementary Figure 7d, bottom row). This behaviour continues for all time points $t < t_{\max}$ until the reaction is completed (Supplementary Figure 7e). It is important to stress that **B** continuously and progressively forms from **A** for all $t < t_{\max}$, with the overall transverse magnetization of **B** comprised of discrete and individual vector elements that all have the same magnitude, but have distinct and unique phase contributions that are given by the instantaneous **A**-spin phase that corresponds to the state of **A** at the time a given **B** magnetization vector element was formed. The total **B** signal is therefore given by the sum of all the individual **B** vector elements that are formed for all infinitesimal time elements dt when $t < t_{\max}$, which is mathematically represented by the integral given by Equation (S3.2). At $t = t_{\max}$, when all of **A** has reacted, the entire ensemble of discrete **B** magnetization vector elements then evolves in unison (Supplementary Figure 7f) under the effects of the **B**-spin Liouvillian and precesses collectively at ω_B, which for this example is zero (*i.e.*, on resonance with the **B** peak), thus each of the individual **B** vector elements remain stationary in the rotating frame (Equation (S3.3)). Plots of these time-domain dynamics (in the absence of T_2 relaxation) are displayed in Supplementary Figures 7g-7j, which show a simultaneous decay (*i.e.*, resulting from the irreversible chemical kinetics) and +50 Hz chemical shift oscillation of **A** for $t < t_{\max}$ (Supplementary Figure 7g, blue trace), as well as the corresponding $\Delta\omega_{AB} = \omega_A - \omega_B$ = +50 – 0 = +50 Hz oscillation of **B** when $t < t_{\max}$ (Supplementary Figure 7g, red trace). At times $t \geq t_{\max}$ post excitation, the irreversible reaction has gone to completion as is indicated by the absence of the **A** signal (Supplementary Figure 7g, blue trace) and the stationary non-oscillatory **B** signal. It is important to note that the integral in Equation (S3.3) sets the initial phase of the subsequent ω_B oscillation when $t \geq t_{\max}$. This point is illustrated further in Supplementary Figure 7h and Supplementary Figure 7i, which were simulated for the same $\Delta\omega_{AB}$ frequency difference of +50 Hz, but at two slightly different k rates (3.60 s^{-1} and 3.53 s^{-1}), resulting in ω_B trajectories that differ by 90°. Supplementary Figure 7j shows the behaviour of the signal of **B** for a non-zero ω_B chemical shift (ω_A = +50 Hz and ω_B = −40 Hz), which results in a multi-frequency oscillation for $t < t_{\max}$ that eventually evolves into a single frequency oscillation at the ω_B frequency when all of **A** has reacted. Supplementary Figures 7k-7m showcase the typical line shapes expected from these off-equilibrium zero-order NMR

kinetics, which were simulated in the fast (Supplementary Figure 7k), intermediate (Supplementary Figure 7l), and slow (Supplementary Figure 7m) exchange regimes. This model is now the starting point for elucidating the effects of plug-like flow before and during FID acquisition, which has the effect of spatiotemporally encoding the reaction kinetics along the spatial coordinate of the NMR coil, whose effects are discussed in the main text.

Supplementary Note 4. **Flowing spins in oscillating magnetic field gradients**.

The following analysis investigates the spin dynamics arising from non-reacting NMR-active nuclei that are simultaneously affected by uniform plug-like flow and different magnetic field gradient waveforms. The combined effects of motion and field gradients on the resulting NMR responses are well understood;[3–5] several examples that are relevant to the EPSI-based kinetic NMR method discussed in the main text, are recapitulated here for the sake of clarity. In all cases, the spin dynamics evolving under the gradient waveforms shown in Supplementary Figure 8 and elsewhere in this Paragraph, are derived assuming plug-like flow conditions characterized by a uniform flow velocity *v*.

(i) A single bipolar gradient phase encodes flow and echoes the effects of stationary spins

Supplementary Figure 8a shows a single bipolar readout gradient that is applied in the same direction as the uniform flow, which is in the *z* direction. The phase evolution accrued for a single spin at an initial position z_0 is determined for each gradient lobe as

$$\phi_1(0 \leq t \leq T_a) = \int_0^{T_a} \gamma G_z(t')z(t')dt' = +G_z\gamma \int_0^{T_a} (z_0 + vt')dt' = +G_z\gamma \left\{z_0[T_a] + \frac{1}{2}v[T_a^2]\right\} \quad \text{(S4.1)}$$

$$\begin{aligned}\phi_2(T_a \leq t \leq 2T_a) &= \int_{T_a}^{2T_a} \gamma G_z(t')z(t')dt' \\ &= -G_z\gamma \int_{T_a}^{2T_a} (z_0 + vt')dt' = -G_z\gamma \left\{z_0[2T_a - T_a] + \frac{1}{2}v[4T_a^2 - T_a]\right\} \\ &= -G_z\gamma \left\{z_0[T_a] + \frac{3}{2}v[T_a^2]\right\}\end{aligned} \quad \text{(S4.2)}$$

$$\phi_1(0 \leq t \leq T_a) + \phi_2(tT_a \leq t \leq 2T_a) = G_z\gamma \left\{z_0[0] + v\left[\frac{1}{2}T_a^2 - \frac{3}{2}T_a^2\right]\right\} = -\gamma G_z v T_a^2 \quad \text{(S4.3)}$$

Therefore, Equation (S4.3) demonstrates that at the end of the second gradient pulse, the contribution of stationary spins (*i.e.*, v independent spins) to the transverse phase is refocused whilst the contributions emanating from moving spins remain. This type of bipolar gradient readout is therefore selective for spins that are moving after a duration of $2T_{\mathrm{a}}$.

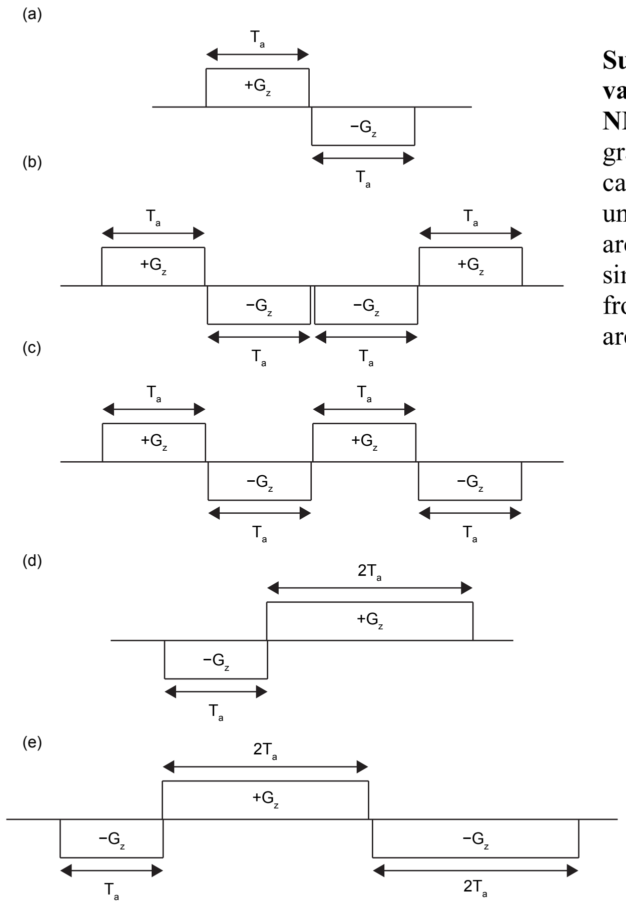

Supplementary Figure 8. Evaluating the effects of various gradient waveforms and uniform flow on the NMR signal phase. Schematic representations of the gradient sequences over which the transverse phase is calculated for a single z_0 position under the effects of uniform flow. As show in the text variants (a), (c), and (d) are not flow compensated – the transverse phase is not simultaneously refocused from contributions originating from static spins and moving spins. By contrast (b) and (e) are flow compensated.

(ii) A symmetric pair of bipolar gradients is flow-compensated

Supplementary Figure 8b shows a pair of symmetrically-placed bipolar gradients, that are again applied along the z direction collinear with the uniform flow. ϕ_1 and ϕ_2 are the same in this case as derived above, so ϕ_3 and ϕ_4 are then:

$$\phi_3(2T_a \leq t \leq 3T_a) = \int_{2T_a}^{3T_a} \gamma G_z(t')z(t')dt'$$

$$= -G_z\gamma \int_{2T_a}^{3T_a} (z_0 + vt')dt'$$

$$= -G_z\gamma \left\{ z_0[3T_a - 2T_a] + \frac{1}{2}v[9T_a^2 - 4T_a] \right\}$$

$$= -G_z\gamma \left\{ z_0[T_a] + \frac{5}{2}v[T_a^2] \right\} \quad \text{(S4.4)}$$

$$\phi_4(3T_a \leq t \leq 4T_a) = \int_{3T_a}^{4T_a} \gamma G_z(t')z(t')dt'$$

$$= +G_z\gamma \int_{3T_a}^{4T_a} (z_0 + vt')dt'$$

$$= +G_z\gamma \left\{ z_0[4T_a - 3T_a] + \frac{1}{2}v[16T_a^2 - 9T_a] \right\}$$

$$= +G_z\gamma \left\{ z_0[T_a] + \frac{7}{2}v[T_a^2] \right\} \quad \text{(S4.5)}$$

All together now,

$$\phi_1(0 \leq t \leq T_a) + \phi_2(T_a \leq t \leq 2T_a) + \phi_3(2T_a \leq t \leq 3T_a)$$
$$+ \phi_4(t3T_a \leq t \leq 4T_a)$$
$$= -\gamma G_z v T_a^2 + 0 + G_z\gamma \left\{ -\frac{5}{2}v[T_a^2] + \frac{7}{2}v[T_a^2] \right\}$$
$$= -\gamma G_z v T_a^2 + \gamma G_z v T_a^2 = 0 \quad \text{(S4.6)}$$

Therefore, the total transverse phase after 2 bipolar readouts (Supplementary Figure 8b) is equal to zero for both stationary spins and spins moving with uniform velocity.

(iii) A pair of bipolar gradients is not flow-compensated

Supplementary Figure 8c shows a pair of bipolar readout gradients, of the kind that would concatenate in an EPSI sequence. The phase accumulated at ϕ_3 and ϕ_4 in this case is:

$$\phi_3(2T_a \leq t \leq 3T_a) = \int_{2T_a}^{3T_a} \gamma G_z(t')z(t')dt'$$

$$= +G_z\gamma \int_{2T_a}^{3T_a} (z_0 + vt')dt'$$

$$= +G_z\gamma \left\{ z_0[3T_a - 2T_a] + \frac{1}{2}v[9T_a^2 - 4T_a^2] \right\}$$

$$= +G_z\gamma \left\{ z_0[T_a] + \frac{5}{2}v[T_a^2] \right\} \quad \text{(S4.7)}$$

$$\phi_4(3T_a \leq t \leq 4T_a) = \int_{3T_a}^{4T_a} \gamma G_z(t')z(t')dt'$$

$$= -G_z\gamma \int_{3T_a}^{4T_a} (z_0 + vt')dt'$$

$$= -G_z\gamma \left\{ z_0[4T_a - 3T_a] + \frac{1}{2}v[16T_a^2 - 9T_a] \right\}$$

$$= -G_z\gamma \left\{ z_0[T_a] + \frac{7}{2}v[T_a^2] \right\} \quad \text{(S4.8)}$$

Adding ϕ_1 to ϕ_4 gives:

$$\phi_1(0 \leq t \leq T_a) + \phi_2(T_a \leq t \leq 2T_a) + \phi_3(2T_a \leq t \leq 3T_a)$$
$$+ \phi_4(t3T_a \leq t \leq 4T_a)$$
$$= -\gamma G_z v T_a^2 + 0 + G_z\gamma \left\{ +\frac{5}{2}v[T_a^2] - \frac{7}{2}v[T_a^2] \right\}$$
$$= -\gamma G_z v T_a^2 - \gamma G_z v T_a^2 = -2\gamma G_z v T_a^2 \quad \text{(S4.9)}$$

Equation (S4.9) shows that a pair of bipolar gradients is not flow-compensated: it refocuses the phase evolution originating from static spins, but moving spins accrue twice the phase as in a single bipolar gradient.

(iv) A gradient echo is not flow compensated

Supplementary Figure 8d shows a conventional gradient echo sequence with the pre-phase and readout gradient applied along the flow direction, *z*. The transverse phase accumulated under the action of the pre-phaser, ϕ_1, is:

$$\phi_1(0 \le t \le T_a) = \int_0^{T_a} \gamma G_z(t')z(t')dt' = -\gamma G_z \int_0^{T_a} (z_0 + vt')dt' = -\gamma G_z \left\{z_0[T_a] + \frac{1}{2}v[T_a^2]\right\} \quad \text{(S4.10)}$$

Similarly, the phase accumulated up to the middle of ϕ_2 (*i.e.*, at the point the stationary spins are refocused) is:

$$\begin{aligned}\phi_2(T_a \le t \le 2T_a) &= \int_{T_a}^{2T_a} \gamma G_z(t')z(t')dt' \\ &= +\gamma G_z \int_{T_a}^{2T_a} (z_0 + vt')dt' = +\gamma G_z \left\{z_0[2T_a - T_a] + \frac{1}{2}v[4T_a^2 - T_a^2]\right\} \\ &= +\gamma G_z \left\{z_0[T_a] + \frac{1}{2}v[3T_a^2]\right\}\end{aligned} \quad \text{(S4.11)}$$

It's clear that although stationary spins produce an echo, the phase contributions from flowing spins are not refocused in the center of the gradient echo. Continuing this analysis for the rest of the reversed gradient, the phase at the end of this gradient is:

$$\begin{aligned}\phi_2(T_a \le t \le 3T_a) &= \int_{T_a}^{3T_a} \gamma G_z(t')z(t')dt' \\ &= +\gamma G_z \int_{T_a}^{3T_a} (z_0 + vt')dt' = +\gamma G_z \left\{z_0[3T_a - T_a] + \frac{1}{2}v[9T_a^2 - T_a^2]\right\} \\ &= +\gamma G_z\{z_0[2T_a] + v[4T_a^2]\}\end{aligned} \quad \text{(S4.12)}$$

The total phase at the end of the sequence is therefore

$$\begin{aligned}\phi_1(0 \le t \le T_a) + \phi_2(T_a \le t \le 3T_a) &= -\gamma G_z \left\{z_0[T_a] + \frac{1}{2}v[T_a^2]\right\} + \gamma G_z\{z_0[2T_a] + v[4T_a^2]\} \\ &= +\gamma G_z \left\{z_0[T_a] + \frac{7}{2}v[T_a^2]\right\}\end{aligned} \quad \text{(S4.13)}$$

(v) A pre-phased bipolar gradient is flow compensated

Supplementary Figure 8e shows a bipolar gradient readout preceded by a pre-phasing period lasting half the length of a single readout gradient (*i.e.*, T_a). In this case, only ϕ_3 needs to be explicitly calculated, since the phase evolution for the first read gradient and pre-phaser is identical to that of a conventional gradient echo (Equation (S4.13)). Therefore,

$$\begin{aligned}\phi_3(3T_a \le t \le 4T_a) &= \int_{3T_a}^{4T_a} \gamma G_z(t')z(t')dt' \\ &= -\gamma G_z \int_{3T_a}^{4T_a} (z_0 + vt')dt' = -\gamma G_z \left\{z_0[4T_a - 3T_a] + \frac{1}{2}v[16T_a^2 - 9T_a^2]\right\} \\ &= -\gamma G_z \left\{z_0[T_a] + \frac{7}{2}v[T_a^2]\right\}\end{aligned} \quad \text{(S4.14)}$$

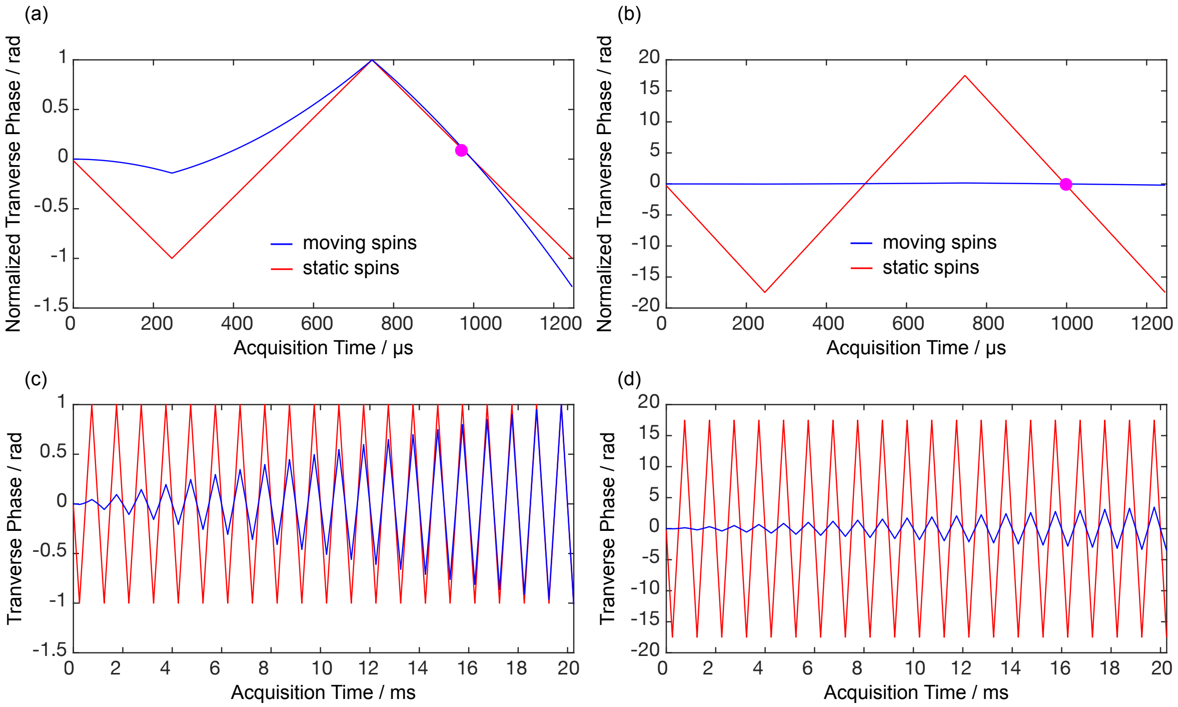

Supplementary Figure 9. Plots of the transverse phase resulting from the action of a pre-phased bipolar readout gradient (shown in Supplementary Figure 8e, which is the basis of an EPSI readout with N_{EPSI} = 1). Plots in the top row show the phase from the static (red) and moving spins (blue) over the course of the three gradients (prephase - (+)-ve gradient - (-)-ve) with T_a = 500 μs and G_z = 26.14 gauss/cm. Plots in the bottom row show the phase evolution over N_{EPSI} = 20 readouts. Data in the left column are normalized, whereas data in the right column are unnormalized. The phase was calculated for z_0 = 0.1 cm and v = 1 cm/s.

ϕ_3 is the transverse phase calculated until the gradient echo is formed in the center of the third gradient pulse. Adding Equation S4.13 to S4.14 gives:

$$\begin{aligned}\phi_1(0 \le t \le T_a) + \phi_2(T_a \le t \le 3T_a) + \phi_3(3T_a \le t \le 4T_a) \\ = +\gamma G_z\left\{z_0[T_a] + \frac{7}{2}v[T_a^2]\right\} - \gamma G_z\left\{z_0[T_a] + \frac{7}{2}v[T_a^2]\right\} = 0\end{aligned} \tag{S4.15}$$

Equation (S4.15) shows that a bipolar gradient applied with a pre-phaser does indeed refocus the phase evolution of both the static and moving spins. The plots in Supplementary Figure 9 show the evolution of both the phase for the static spins (shown in red) and spins with uniform velocity (shown in blue) over the course of one (Supplementary Figure 9a and Supplementary Figure 9b) and 20 (Supplementary Figure 9c and Supplementary Figure 9d) bipolar readout gradients with an initial phasing period lasting half the length of a single readout gradient. The plots in the left column are normalized with respect to the phase accumulated by the static spins in order to better compare their values to those of the moving spins, since the contributions to the phase emanating from the latter are small for small values of t. The magenta circles indicate the time values when the phase for static and moving spins are both equal to zero.

Supplementary Note 5. **Gradient-driven retrieval of spatiotemporally-encoded zero-order kinetics: Derivation and analysis**.

In this section, the mathematical details concerning the derivation of Equations (9-11) of the main text are provided, in addition to a brief analysis of the expressions that describe the spin dynamics of time-resolved spatiotemporally-encoded kinetic NMR spectra. Composing the expression that describes the NMR evolution of product over the course of an EPSI readout begins by multiplying each unique chemical shift term in Equations (S3.2) and (S3.3) by its corresponding gradient-induced frequency shift, which gives for Equation (S3.2),

$$\begin{aligned} \mathrm{S_B}(\mathrm{t} < \mathrm{t_{max}}) \propto & \int_Z \mathrm{M_+^B}(\mathrm{z},\mathrm{t}) \times \exp(\lambda_\mathrm{B}\mathrm{t}) \times \exp\left(i\gamma \int_0^t \mathrm{G}(t') \cdot z(t')dt'\right) \mathrm{dz} \\ & + \int_Z \left\{ \int_0^\mathrm{t} \mathrm{P_{A\to B}}(\mathrm{t}') \times \exp(\lambda_\mathrm{A}\mathrm{t}') \times \exp\left(i\gamma \int_0^{t'} \mathrm{G}(t'') \cdot z(t'')dt''\right) \right. \\ & \left. \times \exp\big(\lambda_\mathrm{B} \cdot (\mathrm{t}-\mathrm{t}')\big) \times \exp\left(i\gamma \int_{t'}^t \mathrm{G}(t'') \cdot z(t'')dt''\right) \mathrm{dt}' \right\} dz \end{aligned} \tag{S5.1}$$

and for Equation (S3.3),

$$\begin{aligned} \mathrm{S_B}(\mathrm{t} \geq \mathrm{t_{max}}) \propto & \int_Z \mathrm{M_+^B}(\mathrm{z},\mathrm{t}) \times \exp(\lambda_\mathrm{B}\mathrm{t}) \times \exp\left(i\gamma \int_0^t \mathrm{G}(t') \cdot z(t')dt'\right) \mathrm{dz} \\ & + \int_Z \left[\left\{ \int_0^{\mathrm{t_{max}}} \mathrm{dt}'\mathrm{P_{A\to B}}(\mathrm{t}') \times \exp(\lambda_\mathrm{A}\mathrm{t}') \times \exp\left(i\gamma \int_0^{t'} \mathrm{G}(t'') \cdot z(t'')dt''\right) \right. \right. \\ & \left. \times \exp\big(\lambda_\mathrm{B} \cdot (\mathrm{t_{max}}-\mathrm{t}')\big) \times \exp\left(i\gamma \int_{t'}^{\mathrm{t_{max}}} \mathrm{G}(t'') \cdot z(t'')dt''\right) \right\} \\ & \left. \times \exp\big(\lambda_\mathrm{B} \cdot (\mathrm{t}-\mathrm{t_{max}})\big) \times \exp\left(i\gamma \int_{\mathrm{t_{max}}}^{\mathrm{t}} \mathrm{G}(t'') \cdot z(t'')dt''\right) \right] \mathrm{dz}, \end{aligned} \tag{S5.2}$$

Simplifying the last term in both equations representing the gradient-induced shifts by exploiting the properties of integrals that have common integration limits and then integrating over t gives the piece-wise expression for the **B** signal contained in Equations (10) and (11) of the main text. These equations (9-11) can then be used to model the experimental kinetic EPSI NMR spectra, of which a representative example is shown in Supplementary Figure 10 for three limiting cases. In Supplementary Figure 10a, the linear flow rate is set to 2 cm/s with a reaction rate $k = 0\ \mathrm{s}^{-1}$, which gives the EPSI NMR spectrum of only the reactant under conditions of continuous flow. The effects of the flow-induced broadening are clearly observed over spatial regions that eventually become vacated of NMR-emitting spins. Supplementary Figure 10b shows the EPSI NMR

spectrum of the off-equilibrium reaction (k = 1 s^{-1}) acquired under stopped-flow conditions, leading to the absence of flow-induced broadening. The flow rate before acquisition was 2 cm/s, which results in the spatiotemporal encoding of the reaction kinetics. Lastly, in Supplementary Figure 10c, both the reaction rate and the flow rate are non-zero over the course of the EPSI readout, which correspond to experimental-like conditions (v = 2 cm/s and k = 1 s^{-1}). Notice how the same degree of flow-induced broadening affects the spectral dimension of the EPSI readout as in Supplementary Figure 10a. Furthermore, the contributions to the **B** signal that arise from reaction of **A** post-excitation are largely absent from these data due to the small value of *k,* leading to signal intensities that are orders of magnitude smaller than the signal intensity arising from equilibrium magnetization formed pre-excitation.

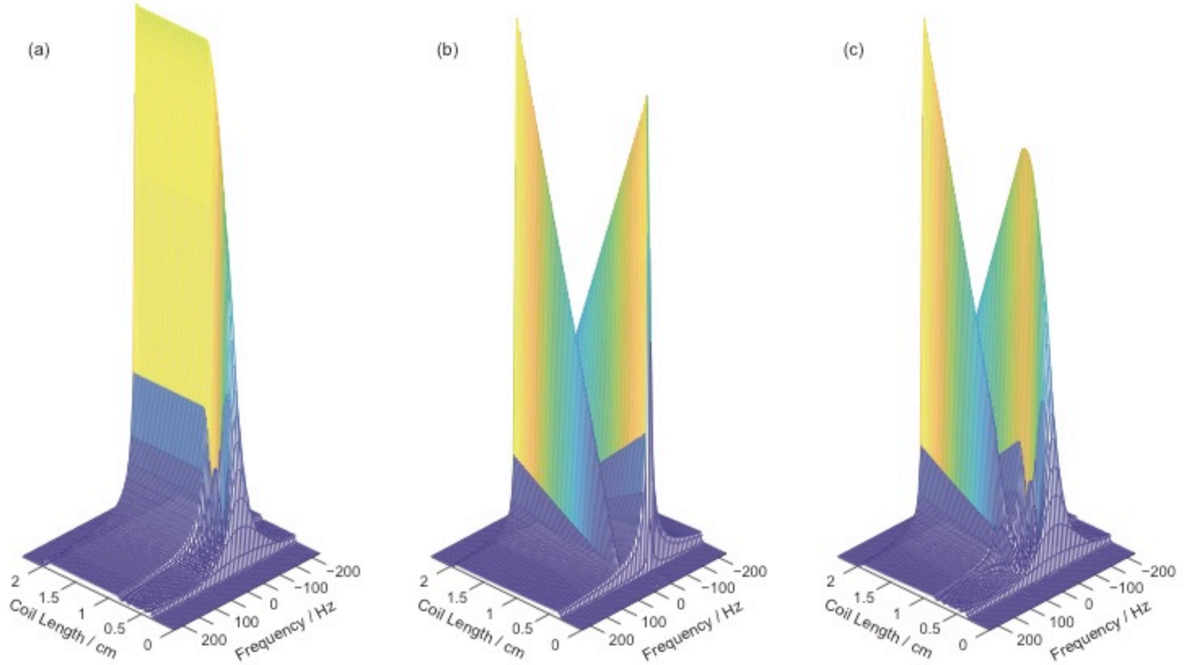

Supplementary Figure 10. **Analytical simulations of EPSI NMR spectra using Equations (9-11) of the main text.** Simulated EPSI NMR spectra assuming (a) $k = 0$ s^{-1}, (b) stopped-flow conditions (with a pre-excitation flow rate of v = 2 cm/s), and (c) non-zero flow- and reaction-rates post-excitation (v = 2 cm/s and k = 1 s^{-1}). The spatially-varying signal intensity is reflective of the zero-order kinetics occurring pre- and post-excitation. A total of 256 gradient echoes were included, each composed of 128 points and lasting 0.5 ms. The image field-of-view was set to 2.3 cm and the gradient amplitude equalled 26.14 G/cm. The chemical shift of the reactant and product were simulated to be $\omega_A/2\pi = -$ 40 Hz and $\omega_B/2\pi$ = +50 Hz, respectively.

Supplementary Note 6. **Examining the effects of different flow profiles on 2D spectrotemporal correlations**

The previous analyses assumed a simple plug flow, devoid of dependence along the axis that is transverse to the flow direction. This section examines the effects of more realistic fluid-flow profiles on the resulting 2D EPSI NMR experiments. Specifically, the accuracy of the 2D spectrotemporal correlations for faithfully encoding the underlying kinetics is investigated when the plug-flow assumption is no longer valid. *In lieu* of a triple-axis gradient system capable of experimentally measuring such profiles, these studies relied on simulations performed using the Ansys's Fluent® fluid dynamics program (https://www.ansys.com/products/fluids/ansys-fluent).[6] This tool was used to calculate flow based on different experimental parameters and conditions, and the flow profiles thus calculated were then used as input for simulating the corresponding NMR experiments and observables.

Supplementary Figure 11 illustrates the various flow profiles that were considered. These included plug flow (Supplementary Figure 11a), laminar flow in a tube for the conditions in Figure 2 but in the absence of the small central capillary used in this study to bring in the reactants (Supplementary Figure 11b), and flow in the presence of the central capillary (Supplementary Figures 11c) for the flow conditions in Figure 2. The flow profiles generated by the Ansys Fluent software led to the transverse velocity profiles shown in the left-most column of Supplementary Figure 11 – all of them calculated under the no-slip (at the walls) assumption. In the presence of chemical kinetics these flow profiles translate into the 2D transverse/longitudinal concentration profiles shown in the second and third left-most columns. The plots on the two right-most columns of Supplementary Figure 11 show the profiles of reactant (A) and product (B), for zero-order kinetic parameters based on Eqs. (1, 2) (main text) and flow parameters as given in the caption.

With this displacement information as background, Supplementary Figure 12 shows EPSI spectra simulated using each of these different fluid-flow profiles (left-most column). In essence these simulations entailed taking the spatial translation operator $\mathcal{T}$ introduced in the main text's theoretical analysis, and making it spatially-dependent along the y-axis as dictated by the numerically calculated flow profile. This translated magnetizations from their initial positions at $t = 0$, by an amount $\delta z(y) = v(y)t$, whose contributions were then suitably weighted in proportion to y in order to account for an integration over the tube's radius. As can be seen from the contour plots of these 2D

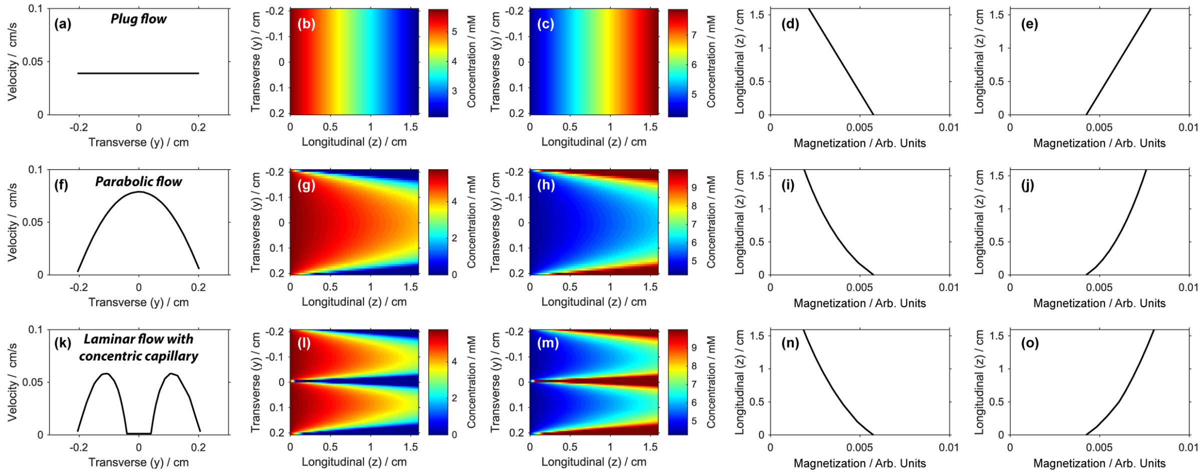

Supplementary Figure 11. **Simulations of plug-like and laminar flow profiles on spectrotemporally-related qualities.** Each row corresponds to a distinct Ansys-generated flow profile; the concentric inflowing capillary / outflowing tube has the dimensions used in the actual experiments. The second and third left-most columns correspond to the reactant and product concentrations, respectively, calculated in the presence of kinetics and for each of the flow profiles. The *y*-axis corresponds to the distance across the reactor tube, which is perpendicular to a flow direction assumed along *z*. The two columns on the right correspond to the magnetization expected from each flow profile as a function of *z* position, upon an EPSI readout. Concentrations/magnetizations for each row were simulated based on Eqs. (1,2) (main text) and the following zero-order kinetic parameters: $[R]_0$ = 10 mM, $[E]_0$ = 7.5 µM, k_{cat} = 11.8 s^{-1}, and t_0 = 48 s.

spectra and from their corresponding 1D spectral traces (extracted at the horizontal green lines and displayed in middle column of Supplementary Figure 12), the spectral dimension of the experiment –*i.e.*, peak shapes and peak locations– remains unchanged under the different flow profiles. Departure from plug flow, however, introduces a distortion from ideality in the peak intensities associated to the various *z*-positions (Supplementary Figure 12, right-hand column). This reflects the distortions that each flow profile makes on the position/reaction-time dependence. In plug flow this is simply linear (Supplementary Figure 12a, right-most column) and peak intensities allow one to read out the correct k_{cat} and t_0 values from a linear fitting. The position-dependent intensities that result for the remaining conditions are no longer proportional to reaction time (Supplementary Figure 11, middle columns); this leads to a curvature in the peak intensities *vs* position. For the parabolic flow, using the predicted position-dependent intensities to obtain a fit leads to a good linear relation, but to an apparent reaction rate that is slightly larger than what was utilized as ground truth. Both the quality of the fit and of the value of the kinetic rate improve if the flow profile considers the central capillary used in our experiments (Supplementary Figure 12, left-most column). These realistic numerical simulations, coupled to the fact that the fitted kinetic parameters determined from the spectra in Figures 2 and 4 of the main text are in good agreement with both control experiments and literature values, justifies –at

least under the assayed flow conditions– the adoption of the plug-like approximation in the analysis of this type of experiments.

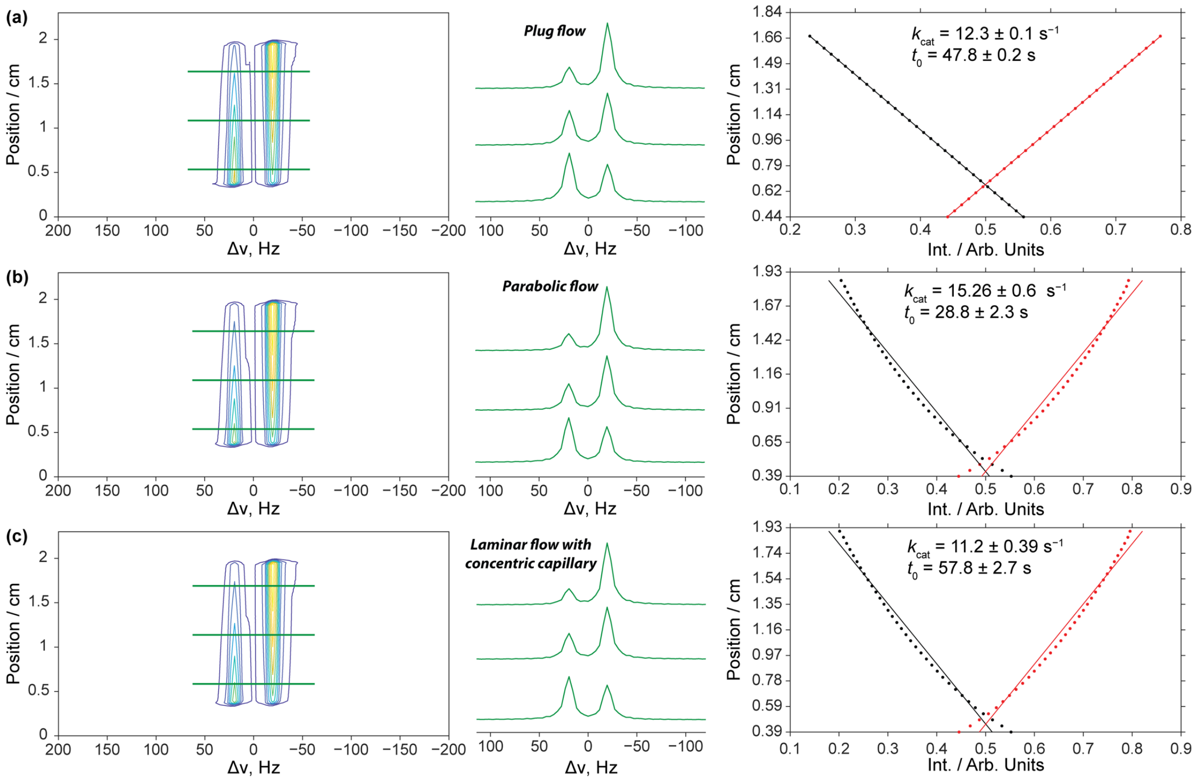

Supplementary Figure 12. **EPSI NMR spectral line shape calculations/derivations based on Ansys Fluent fluid dynamic simulations, for each of the three flow profiles introduced in Supplementary Figure 11.** The simulated EPSI NMR spectra on the left arise from a zero-order reaction (Eqs. 1,2) assuming $[R]_0$ = 10 mM, $[E]_0$ = 7.5 uM, k_{cat} = 11.8 s^{-1} and t_0 = 48 s. The fluid flow profiles input into these EPSI NMR simulation code are as shown in the left-most column of Supplementary Figure 11 and assumed an average flow velocity of 0.33 mL/s (matching the conditions used in Figure 2 of the main text). Shown in the central column are selected spectral profiles extracted at the indicated positions for each of these flow profiles. The right-most column shows with dotted lines the relative intensity changes of reactants and products under the different flow conditions, as quantified from the EPSI spectra in the left-most column. Also indicated in these graphs by straight lines are linear fits of each intensity data set, after invoking the plug-flow assumption. Not surprisingly there is an excellent agreement of the apparent k_{cat} and t_0 for the top-most row, and a very good agreement of the apparent k in the bottom double-parabolic flow profile, as well.

Supplementary References:

(1) Callaghan, P. T. *Principles of Nuclear Magnetic Resonance Microscopy*; Oxford: Clarendon Press, 1993.

(2) Tal, A.; Frydman, L. Single-Scan Multidimensional Magnetic Resonance. *Prog. Nucl. Magn. Reson. Spectrosc.* **2010**, *57* (3), 241–292.

(3) Stejskal, E. O. Use of Spin Echoes in a Pulsed Magnetic-Field Gradient to Study Anisotropic, Restricted Diffusion and Flow. *J. Chem. Phys.* **1965**, *43* (10), 3597–3603.

(4) Walton, J. H.; Conradi, M. S Flow Velocity Measurement With AC Gradients. *Magn.*

Reson. Med. **1987**, *4* (3), 274–281.
(5) Caprihan, A.; Fukushima, E. Flow Measurements by NMR. *Phys. Rep.* **1990**, *198* (4), 195–235.
(6) Ansys Fluent, Release R2. https://www.ansys.com/products/fluids/ansys-fluent
